# Cytoplasmic sequestration of the RhoA effector mDiaphanous1 by Prohibitin2 promotes muscle differentiation

**DOI:** 10.1038/s41598-019-44749-4

**Published:** 2019-06-05

**Authors:** Amena Saleh, Gunasekaran Subramaniam, Swasti Raychaudhuri, Jyotsna Dhawan

**Affiliations:** 1 Institute for Stem Cell Science & Regenerative Medicine, Bangalore, Karnataka 560065 India; 2 Council of Scientific & Industrial Research -Centre for Cellular & Molecular Biology, Hyderabad, Telangana 500007 India; 3 Manipal Academy of Higher Education, Manipal, Karnataka 576104 India; 4 Department of Physiology, Anatomy and Genetics, University of Oxford, Oxford, OX1 3PT UK

**Keywords:** RHO signalling, Cellular imaging

## Abstract

Muscle differentiation is controlled by adhesion and growth factor-dependent signalling through common effectors that regulate muscle-specific transcriptional programs. Here we report that mDiaphanous1, an effector of adhesion-dependent RhoA-signalling, negatively regulates myogenesis at the level of Myogenin expression. In myotubes, over-expression of mDia1ΔN3, a RhoA-independent mutant, suppresses Myogenin promoter activity and expression. We investigated mDia1-interacting proteins that may counteract mDia1 to permit Myogenin expression and timely differentiation. Using yeast two-hybrid and mass-spectrometric analysis, we report that mDia1 has a stage-specific interactome, including Prohibitin2, MyoD, Akt2, and β-Catenin, along with a number of proteosomal and mitochondrial components. Of these interacting partners, Prohibitin2 colocalises with mDia1 in cytoplasmic punctae in myotubes. We mapped the interacting domains of mDia1 and Phb2, and used interacting (mDia1ΔN3/Phb2 FL or mDia1ΔN3/Phb2-Carboxy) and non-interacting pairs (mDia1H + P/Phb2 FL or mDia1ΔN3/Phb2-Amino) to dissect the functional consequences of this partnership on Myogenin promoter activity. Co-expression of full-length as well as mDia1-interacting domains of Prohibitin2 reverse the anti-myogenic effects of mDia1ΔN3, while non-interacting regions do not. Our results suggest that Prohibitin2 sequesters mDia1, dampens its anti-myogenic activity and fine-tunes RhoA-mDia1 signalling to promote differentiation. Overall, we report that mDia1 is multi-functional signalling effector whose anti-myogenic activity is modulated by a differentiation-dependent interactome. The data have been deposited to the ProteomeXchange with identifier PXD012257.

## Introduction

Cooperation between intrinsic transcriptional programs and extrinsic signalling underlies cell fate choices during development. In skeletal muscle, differentiation is regulated by Muscle Regulatory Factors (MRFs) - MyoD, Myf5, Myogenin (MyoG) and MRF4, whose orchestrated expression and activity governs myogenic gene expression. In embryonic progenitors, MyoD and Myf5 function as lineage determinants regulating the early stages of myogenesis, whereas MyoG and MRF4 function as differentiation factors to promote the later stages of myogenesis and fusion into contractile multinucleated cells^1^. *In vitro*, myoblasts (MB) proliferate when cultured in mitogen-rich media and express MyoD, which binds several myogenic promoters genome-wide^2^ but is kept in a transcriptionally incompetent state by growth factor-dependent post-translational modifications (PTMs)^3,4^. Upon removal of mitogens, MyoD’s transcriptional activity is de-repressed^5–7^, its key transcriptional target Myogenin (MyoG) is induced, and a downstream cascade of muscle-specific genes is activated^8,9^; MB then irreversibly exit the cell cycle and fuse to form syncytial terminally differentiated myotubes (MT)^10–12^. While several signalling pathways that regulate differentiation are known, the multiplicity of downstream effectors and mechanisms by which they channel control of muscle-specific genes is incompletely understood.

Mechano-chemical cues converge with signalling by soluble factors such as Insulin-like Growth Factors (IGFs) to regulate the small GTPase RhoA. RhoA transduces IGF and adhesion-mediated signals to control cytoskeletal dynamics that in turn impact gene expression^13^. Ectopic expression of RhoA in proliferating MB enhances actin stress-fiber formation and induces the expression of differentiation-specific proteins MyoG, p21 and Troponin T^14,15^. Although RhoA activity is required for initial induction of myogenesis^16^, its activity must be down-regulated before myoblast fusion to promote fusion and differentiation^17–20^. Thus, RhoA signalling mediates the effects of extra-cellular stimuli to regulate myogenesis in a stage-specific manner.

Signalling networks may have constitutive as well as state-specific components. The RhoA network consists of several downstream effectors^21,22^, of which mammalian Diaphanous1 (mDiaphanous1 or mDia1) and Rho-associated kinase (ROCK) collectively regulate focal adhesion and stress fiber formation^23–28^. However mDia1, but not ROCK, mediates the effects of RhoA on MyoD expression^14^. mDia1 coordinates the dynamics of both actin filaments and microtubules^29^ and links cytoskeletal rearrangements to transcriptional control^28,30–32^. In proliferating MB, mDia1 transduces RhoA signals to regulate MyoD expression by differentially modulating the activity of Serum Response Factor (SRF) and β-Catenin^33^. However, signals emanating from the mDia1 signalling node in differentiated MT are unknown.

In this study, we probed the potential mediators of mDia1 function in myogenic cells using two screening methods to search for interacting partners. We report the interactome of this RhoA effector in MB and MT, and delineate the role of a novel myotube-specific mDia1-interacting partner Prohibitin2 (Phb2) in regulation of MyoG expression. While Diaphanous (Dia) is known to promote myoblast fusion in flies during myofibrillogenesis^34,35^, a role for mDia1 in mammalian myofibers is less well established.

The newly identified mDia1-interacting partner Phb2 (also known as Repressor of Estrogen Activity), is a multi-functional protein^36,37^, reported to regulate Estrogen Receptor alpha (ERα)-mediated transcription^38–40^, CP2c-mediated transcription^41^ and muscle differentiation^42,43^. We map the domains that mediate mDia1-Phb2 interaction, identify additional signalling proteins as partners, and investigate the consequences of this interaction in regulating MyoD and MyoG expression. In summary, we report a new function for mDia1 in regulation of muscle differentiation and protein partners that modulate this role. Our findings suggest that mDia1 plays a role in maintaining homeostatic mechanisms downstream of RhoA, with additional differentiation-dependent roles that require modulation by stage-specific interacting proteins.

## Results

### Identification of novel interacting partners of mDia1 reveals Phb2, a multi-functional transcriptional regulator

**Figure 1:**
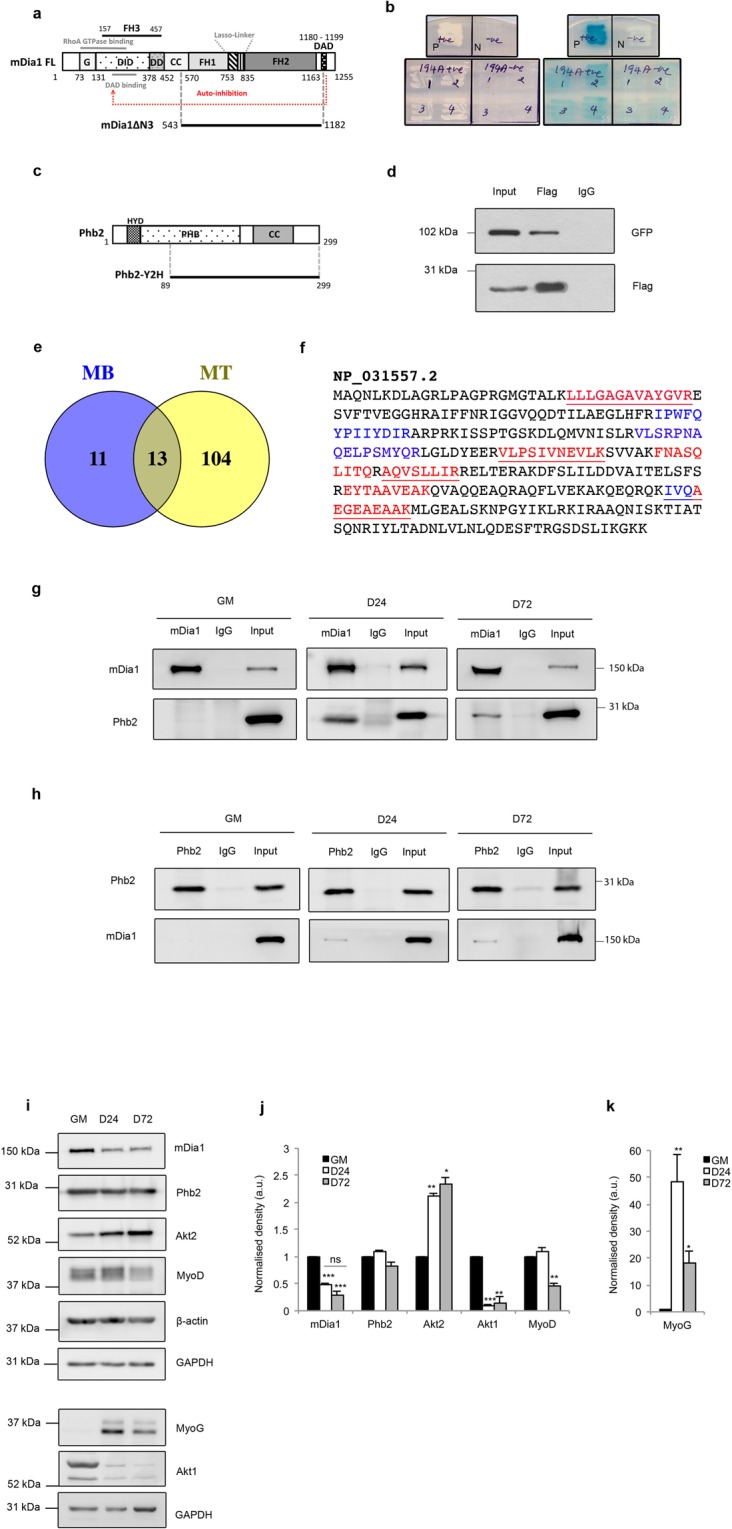
Prohibitin2, a novel mDia1-interacting protein, associates with mDia1 in myotubes. (**a**) Domain structure of full-length (FL) mDia1 and constitutively active mDia1 mutant, mDia1ΔN3. Grey lines indicate RhoA and DAD binding regions. G-GTPase binding domain, DID-Diaphanous Inhibitory Domain, Dimerisation Domain (DD), Coiled Coil (CC), FH1, FH2, FH3-Formin Homology domains, DAD-Diaphanous Auto-inhibitory Domain. Start positions of domains are depicted. (**b**) Phb2 identified as mDia1-interacting protein in a yeast two-hybrid screen. PJ69-4A was co-transformed with Phb2-AD and mDia1ΔN3-BD (positive GAL4 reconstitution) or empty-BD (negative GAL4 reconstitution) and four colonies per reconstitution were screened for *ADE2* and *LacZ* reporters on −Trp/−Leu/−Ade and −Trp/−Leu + X-Gal plates respectively. Growth indicates *ADE2* induction and blue pigmentation indicates *LacZ* induction. Positive control “P”- *Drosophila* Batman-AD and GAGA factor-BD, negative control “N”- empty-AD and empty-BD. Trp-Tryptophan, Leu-Leucine, Ade-Adenine. AD-Activation domain, BD-binding domain. (**c**) Domain structure of Phb2 FL and Phb2-Y2H. HYD-Hydrophobic region, PHB-Prohibitin domain, CC-Coiled coil domain. (**d**) Co-IP of flag-tagged Phb2-Y2H and GFP-tagged mDia1ΔN3 to confirm the interaction. HEK293T, co-transfected with mDia1ΔN3 and Phb2-Y2H, and pulled down with anti-Flag antibody. IP product was run on two different gels 8% and 12% for detecting with anti-GFP and anti-Flag antibodies respectively and these blots were processed in parallel. The blot probed with anti-Flag antibody represented here was cut prior to processing for western blotting. Cropped blot for GFP has been shown here whereas full-length GFP blot is presented in Supplementary Fig. S6. (**e**) LC-MS/MS analysis of mDia1-interacting proteins in myoblasts (MB) and myotubes (MT) in differentiation medium (DM) for 72 hours. Venn diagram represents the number of proteins that bind mDia1 in MB or MT or both MB and MT. (**f**) Phb2 peptides identified in MT lysates by LC-MS/MS analysis of mDia1 IP proteins. Phb2 aa sequence (NCBI Reference Sequence # NP_031557.2) showing peptides identified in first (red), second and third (blue) and all three (underlined) biological replicates. (**g**,**h**) Reciprocal IP of endogenous mDia1 and Phb2 to identify stage-specific interaction. Lysates from proliferating MB (GM), MT in DM for 24 (D24) and 72 (D72) hours were harvested and subjected to IP with anti-mDia1 (**g**) or anti-Phb2 (**h**) antibodies. IP samples were loaded on different gels, blots were cut and processed in parallel, using same conditions of antibody incubation and exposure time during developing. (**i**) Western blot showing the expression profile of mDia1 and Phb2 in GM, D24, and D72 lysates. All the lysates were run on a single gel, blots were cut and probed for mDia1, Phb2, Akt2, MyoD, β-actin and GAPDH. The same lysates were run on a different gel, blots were cut and probed for MyoG, Akt1 and GAPDH. (**j**,**k**) Bar diagram represents the densitometric quantification of western blots shown in (i) *p < 0.05, **p < 0.01, ***p < 0.001 when compared to GM, n = 3. a.u. -arbitrary units. Numbers represent aa position (**a**,**c**). Corresponding sizes are indicated in kDa. ns-not significant.

Previously we showed that mDia1 regulates the expression of MyoD in proliferating MB, by modulating two different transcription factors (TFs)- SRF and T-Cell Factor (TCF)^33^. To probe the mechanisms by which mDia1 functions, we identified its interacting partners using a yeast two-hybrid (Y2H) screen. Full-length (FL) mDia1 is auto-inhibited in the absence of active RhoA signalling^23,44^. To circumvent the requirement for RhoA activation in yeast we used mDia1ΔN3 (543–1192aa), a RhoA-independent constitutively active mutant of mDia1 lacking the Rho-binding domain (RBD)^23^ (Fig. 1a). mDia1ΔN3 fused to GAL4 DNA-binding domain (mDia1ΔN3-BD) was used as bait, while a Matchmaker mouse cDNA library fused to GAL4 activation domain (AD) (Clonetech), served as prey. Putative interacting proteins for mDia1 were selected based on the induction of expression of two reporters– *ADE2* and *LacZ*. Phb2 was identified as one of 8 mDia1-interacting proteins in this Y2H screen (Fig. 1b). Profilin1 (Pfn1), a known partner of mDia1 involved in actin nucleation^24^ was also recovered, validating the screening strategy (Supplementary Fig. S1). Other proteins identified were all members of membrane-cytoplasmic signalling families: Niemann Pick type C2 (Npc2), Cadherin11 (Cdh11), Leukocyte receptor cluster (LRC) member8 (Leng8), Growth receptor bound protein 2 (Grb2), Protein-kinase, interferon-inducible double stranded RNA-dependent inhibitor repressor of P58 (Prkrir) and Cytochrome c1 (Cyc1) (Supplementary Fig. S1 and Table S1). Phb2 was selected for further studies as this protein has been reported to regulate MyoD function in C2C12 MB^42,43^. Phb2-Y2H, a flag-tagged construct encoding the partial Phb2 clone (aa 89–299) recovered in the Y2H screen (Fig. 1c), was used for co-immunoprecipitation (IP) to validate the mDia1-Phb2 interaction in mammalian cells. IP with anti-flag in HEK293T cells co-expressing GFP-tagged mDia1ΔN3 and Phb2-Y2H, resulted in co-IP of mDia1ΔN3 (Fig. 1d), confirming that ectopically expressed mDia1 and Phb2 can interact in mammalian cells.

### LC-MS/MS analysis of mDia1-interacting proteins in MB and MT

To assess the range of mDia1-interacting proteins in muscle cells, we performed LC-MS/MS analysis of mDia1-co-IPs from two stages: proliferating MB and MT in differentiation medium (DM) for 72 hours. mDia1 was identified in both MB and MT, confirming successful immunoprecipitation from both states (Supplementary Table S2). Notably, Phb2 was identified as an mDia1-interacting protein specifically in MT in all three replicates (Supplementary Table S4), further validating the mDia1-Phb2 interaction noted in the Y2H analysis. Phb2 peptides identified by mass spectrometry are shown in Fig. 1f. 13 proteins were commonly associated with mDia1 in both MB and MT. 11 additional mDia1-interacting proteins were exclusively detected in MB and 104 were found only in MT (Fig. 1e). These proteins were reproducibly detected in three independent biological replicates of endogenous mDia1 IP-LC-MS/MS analysis. mDia1-interacting proteins common to MB and MT and specific to MB or MT are listed in Supplementary Tables S2–S4 respectively. We used REVIGO^45^ for gene ontology (GO) analysis of mDia1-interacting proteins (Supplementary Fig. S2). The altered interactome in the two stages suggests that mDia1 function may differ during myogenesis, with an expanded role in MT.

STRING analysis of mDia1-interacting proteins identified clusters of interacting proteins in both states (Supplementary Fig. S3). Networks of mDia1-interacting proteins common to MB and MT or specific to either MB or MT are shown, highlighting, MT-specific networks of proteasomal proteins (red), metabolic enzymes (blue) and mitochondrial proteins (black). The altered mDia1 interactome suggests stage-specific changes in effector function during myogenesis. Since Phb2 has been previously implicated in myogenic differentiation^42,43^, we delineated the consequences of its interaction with mDia1 in detail.

### Endogenous mDia1 and Phb2 interact during myoblast differentiation but not in proliferation

To further characterise the timing of mDia1-Phb2 interaction in C2C12, we performed IP in MB (GM-Growth medium) and MT maintained in DM for 24 (D24), or 72 (D72) hours. Proliferating MB differentiate when cultured in low serum, and fuse to form MT^7,46^. IP using anti-mDia1 antibody showed that endogenous Phb2 was specifically co-immunoprecipitated with mDia1 in early and late MT (D24, D72), but not in MB (GM) (Figs 1g and 4c), validating the Y2H and proteomic analysis. A reciprocal experiment using anti-Phb2 antibody confirmed the specificity of the interaction with mDia1 only in MT (D24, D72) and not in MB (Fig. 1h).

**Figure 2:**
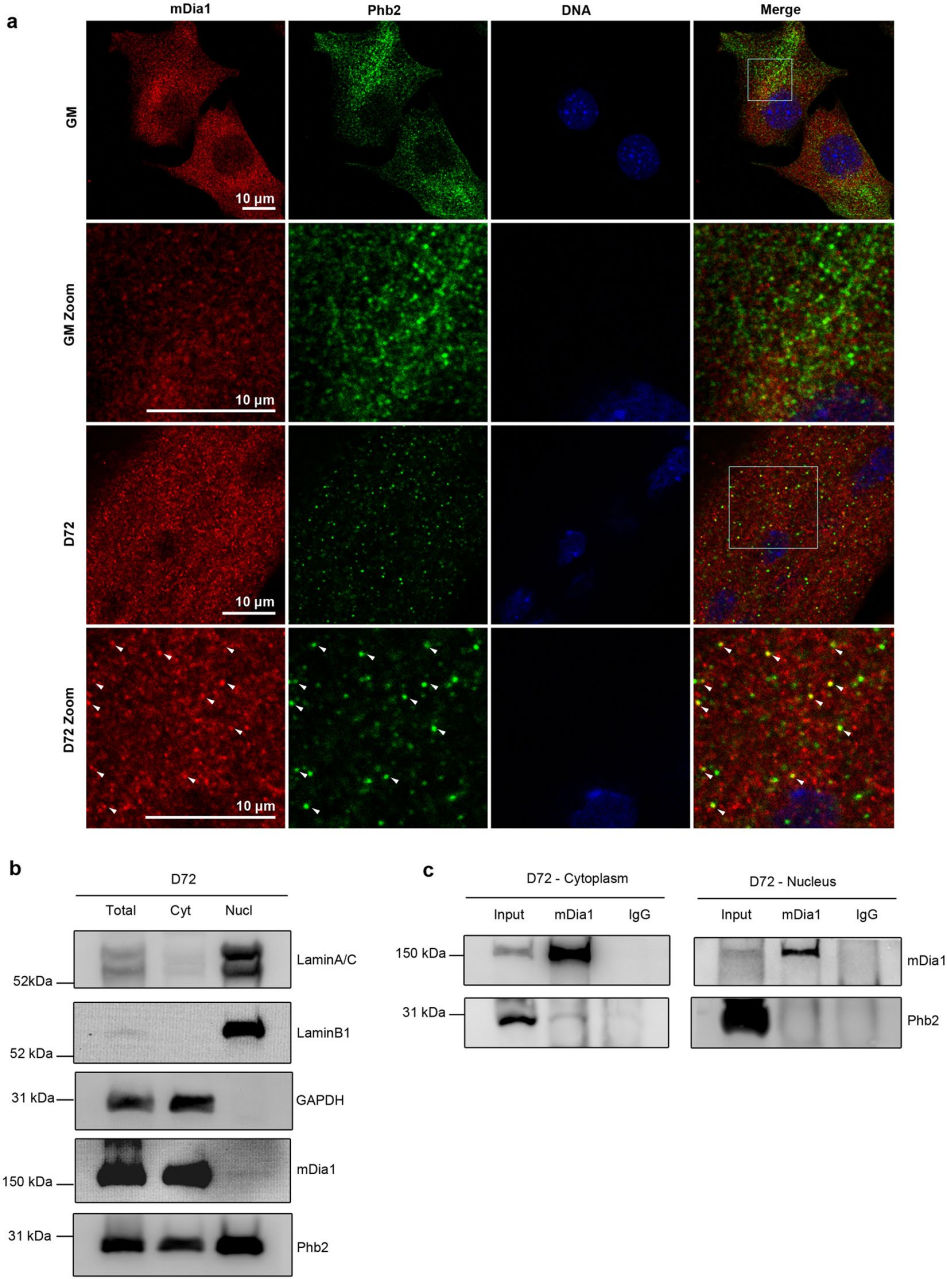
mDia1 interacts with Phb2 in the cytoplasm of myotubes. (**a**) Immunostaining of endogenous mDia1 and Phb2 during proliferation (GM) and differentiation (D72) to detect colocalisation. The white boxes indicate the zoomed regions. Arrows indicate colocalised puncta. Confocal images were acquired using Leica TCS SP8 confocal microscope. (**b**) Purity of cytoplasmic and nuclear fractions of MT (D72). Cytoplasmic and nuclear extracts were prepared from D72 MT, followed by analysis by western blotting with antibodies against cytoplasmic GAPDH, nuclear LaminA/C and LaminB1 to determine the purity of the fractions. Distribution of mDia1 and Phb2 was detected by western blotting using respective antibodies. Lysates were loaded on a single gel, the blot was cut prior to processing in parallel for western blotting. (**c**) IP of mDia1 in cytoplasmic and nuclear extracts to detect localisation of associated Phb2. Cytoplasmic and nuclear extracts were prepared from D72 MT and subjected to IP using anti-mDia1 antibody, followed by detection of Phb2. IP products from cytoplasmic and nuclear extracts were loaded on a single gel, the blot was cut and probed for mDia1 and Phb2 in parallel. Cyt- Cytoplasm, Nucl-nucleus.

**Figure 3:**
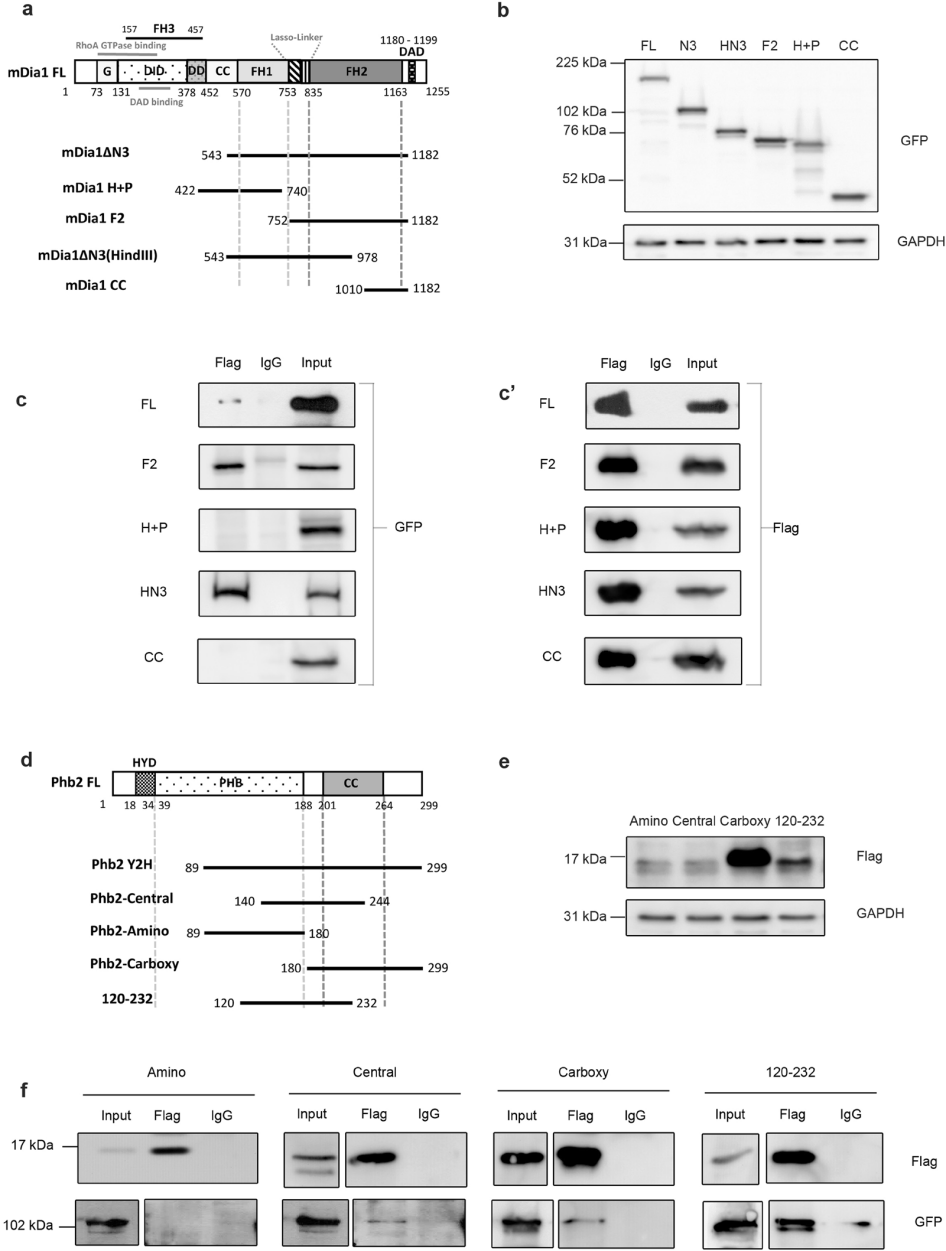
Mapping interaction domains on mDia1 and Phb2. (**a**) Schematic for mouse mDia1 truncation mutants. (**b**) Western blot to detect expression level of mDia1 mutants. Lysates from HEK293T transfected with mDia1 mutants were probed using anti-GFP antibody. GAPDH was used as a loading control. All the lysates were run on a single gel, the blot was cut and processed in parallel for detection of GFP-tagged mDia1 mutants and GAPDH. (**c**,**c**’) Co-IP of mDia1 mutants and Phb2 to map interaction domains. HEK293T cells were transfected with Phb2-Y2H and various mDia1 mutants, followed by IP with anti flag antibody. IP products were run on different gels, the blots were cut and processed in parallel for detection of interacting mutants. (**d**) Schematic for mouse Phb2 truncation mutants. (**e**) Western blot to detect expression of Phb2 mutants. Lysates of HEK293T transfected with Phb2 mutants were loaded on a single gel, the blot was cut and analysed in parallel using anti-flag and anti-GAPDH antibodies. GAPDH was used as a loading control. (**f**) Co-IP of Phb2 mutants and mDia1 to map interaction domains. Lysates from HEK293T cells co-transfected with various Phb2 mutants and mDia1ΔN3 were subjected to IP using anti-flag antibody. IP samples were run on different gels, the blots were cut and processed in parallel for detection with anti-GFP and anti-Flag antibodies under same conditions of detection. Different gels with 12% and 8% were used for the flag and GFP blots respectively. The represented cropped input lanes of Phb2-Amino, Central, Carboxy and 120–232 in the GFP blot show lower exposure of the inputs run along with the corresponding IP samples in the same gel whereas the cropped input lanes from Central, Carboxy and 120–232 in flag blot represent higher exposure of the inputs run along with the corresponding IP samples in the same gel. The numbers represent aa positions (**a**,**d**).

**Figure 4:**
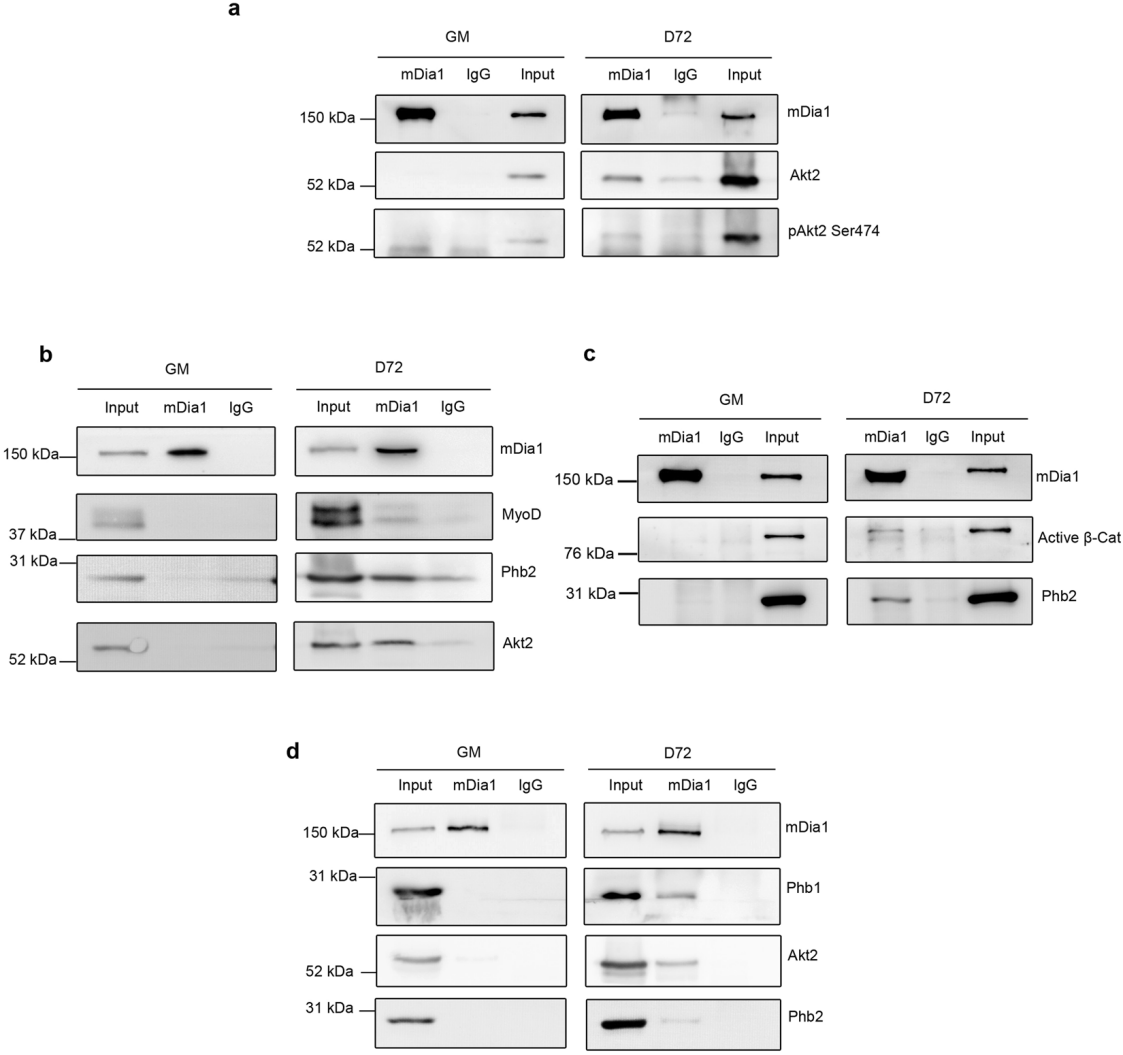
mDia1 co-immunoprecipitates differentiation markers MyoD, active β-Catenin and pAkt2 Ser474 along with Phb2 during differentiation. C2C12 cells cultured under growth conditions (GM) or differentiated for 72 (D72) hours were lysed and subjected to IP with anti-mDia1 antibody, and analysed by western blotting using respective antibodies. (**a**) Co-IP of mDia1 with Akt2 and pAkt2 Ser474 in MT. IP samples were loaded on different gels, cut and processed in parallel for detection of mDia1, Akt2 and pAkt2 Ser474. (**b**) Co-IP of MyoD, Phb2 and Akt2 by mDia1 in MT. (**c**) Co-IP of active β-Catenin and Phb2 by mDia1 in MT. (**d**) IP of Phb1, Akt2 and Phb2 by mDia1 in MT. Act β-Cat- Active β-Catenin. For the blots shown in (**b**–**d**) the IP samples for MB and MT were run on a single gel, the blot was cut and processed in parallel for detection using the respective antibodies under the same conditions. Uncropped version of the blots represented in (**b**–**d**) are shown in Supplementary Fig. S6.

To establish the expression profile of mDia1 and Phb2 we performed western blotting. The differentiation status of cultures at each time point was established by analysing the expression of MyoD, MyoG, Akt2 and Akt1 (Fig. 1i–k). Consistent with their roles, MyoG and Akt2 expression increased during differentiation, while MyoD and Akt1 expression decreased^47,48^. Of the interacting partners, expression of Phb2 remained unchanged, whereas the expression of mDia1 decreased during differentiation. Thus, despite expression in both MB and MT, mDia1 and Phb2 interact in a stage-specific fashion only in MT. We assessed the possibility of altered phosphorylation of known phosphorylation sites, Tyr128 and Tyr248 on Phb2^37,49^ in promoting their interaction (Supplementary Fig. S4). There was no change in the levels of pPhb2 Tyr128 or pPhb2 Tyr248 during differentiation. Thus, while other residues on Phb2 or mDia1^50^ might be modified, the differentiation-specific signalling mechanisms that promote their interaction in MT appear independent of Phb2 Tyr128 or Tyr 248 phosphorylation.

### mDia1 and Phb2 co-localise in cytoplasmic punctae during differentiation

To identify the location of mDia1-Phb2 interaction we performed immunostaining of mDia1 and Phb2 in MB (GM) and MT (D72) (Fig. 2a). Colocalisation of mDia1 and Phb2 was seen in cytoplasmic puncta in MT, but not in MB. The immunostaining was specific to mDia1 and Phb2 as evidenced by reduced fluorescence intensity in mDia1 and Phb2 knockdown myoblasts respectively, and validated by western blotting (Supplementary Fig. S5). Further, IP with anti-Phb2 antibody used for the colocalisation analysis also co-immunoprecipitated mDia1 only in MT, confirming the specificity of the anti-Phb2 antibody (Supplementary Fig. S5). To biochemically validate the location of mDia1-Phb2 interaction, cytoplasmic and nuclear fractions were isolated from D72 MT and their purity verified by markers (Fig. 2b). mDia1 was predominantly cytoplasmic, whereas Phb2 was found in both nuclear as well as cytoplasmic fractions. Anti-mDia1 immunoprecipitation of cytoplasmic and nuclear fractions from MT showed that Phb2 was co-immunoprecipitated specifically by the cytoplasmic pool of mDia1 (Fig. 2c). Taken together, these findings indicate that mDia1 associates with Phb2 in cytoplasmic puncta in MT.

### Mapping of interaction domains on both mDia1 and Phb2

To map the interaction domains on mDia1 and Phb2 we used GFP-tagged mDia1 truncation mutants^23,33^ (Fig. 3a) and flag-tagged Phb2 truncation mutants respectively (Fig. 3d). mDia1 has an N-terminal Rho Binding region including a GTPase binding region (G) and Diaphanous Inhibitory Domain (DID), a Dimerisation Domain (DD), Coiled Coil (CC), three central Formin homology (FH) domains FH1, FH2 and FH3, a flexible lasso-linker region between FH1-FH2 and a C-terminal Diaphanous Autoregulatory Domain (DAD)^51–54^ (Fig. 3a). In the absence of RhoA signalling, mDia1 is kept auto-inhibited via intra-molecular interactions of its DID and DAD domains^23,55–59^. Signalling from RhoA leads to release of auto-inhibition, while deletion of aa 1–542 including the Rho binding region, yields a constitutively active form of mDia1, mDia1ΔN3^23^.

Phb2 contains an N-terminal hydrophobic trans-membrane alpha helix (aa 18–34) (HYD), a central Prohibitin (PHB) domain (aa 39–201) and a C terminal Coiled coil (CC) domain (aa 188–264)^60^ (Fig. 3d). Human and mouse Phb2 proteins are 100% identical. Other important sequences include a positively charged N terminal leader sequence (aa 1–50) that functions as a mitochondrial targeting sequence (MTS) in both human and mouse Phb2 and a nuclear localisation signal at the C terminal in human Phb2^61,62^. Putative nuclear localisation signal (aa 86–89) and nuclear receptor box have been predicted for mouse Phb2^62^. Flag-tagged truncation mutants of Phb2 were generated to span aa 89–299 of Phb2 FL (full-length), the region recovered in the Y2H screen (Fig. 3d).

To map the Phb2-interacting region of mDia1, HEK293T cells were co-transfected with flag-tagged Phb2-Y2H (aa 89–299) and different mDia1 truncation mutants (GFP-tagged). mDia1 mutants expressed at relatively equal levels whereas the Phb2-Carboxy mutant expressed at a higher level than the other Phb2 mutants (Fig. 3b,e respectively). Co-IP using anti-flag antibody was followed by detection with anti-GFP antibody (Fig. 3c,c’). mDia1ΔN3, mDia1F2, mDia1ΔN3(HindIII) interacted with Phb2-Y2H, whereas mDia1H + P and mDia1CC did not. This analysis indicates that mDia1 binds Phb2-Y2H via aa 752–978, which maps to the lasso-linker region and a portion of the FH2 domain which partially includes the FH2 motif (aa 946–1010)^53^. Conceivably, binding of Phb2 to this region (aa 752–978) might regulate mDia1’s activity.

Reciprocally, to map the mDia1-interacting regions of Phb2, HEK293T cells were co-transfected with different flag-tagged Phb2 truncation constructs and mDia1ΔN3-GFP, followed by IP with anti-flag antibody (Fig. 3f). Phb2-Central (aa 140–244), Carboxy (aa 180–299), and 120–232 (aa 120–232) region bound to mDia1, but the Amino (aa 89–180) region did not. Thus, the minimal mDia1-interacting region of Phb2 maps to aa 180–232. This region includes a small portion of the PHB domain and almost half of the coiled coil domain and lies within aa 120–232 region of Phb2. The region aa 120–232 of human PHB2 has been shown to contain overlapping binding sites for MyoD, Akt and ERα (aa175–198)^38,42^ (Supplementary Table S5). Since mouse and human Phb2 proteins are 100% identical, we infer that the binding of mouse mDia1 to Phb2 could compete with the binding of Phb2 to Akt/MyoD/ERα. Taken together, these domain-mapping studies suggest the possibility that Phb2 may regulate gene expression by forming mutually exclusive interactions with key TFs and signalling effectors.

### Phb2 forms a complex with mDia1 and pro-myogenic proteins during differentiation

To determine whether mDia1 and Phb2 form additional interactions with known muscle transcriptional regulators, we pulled down mDia1 and probed for co-immunoprecipitation of Akt2, MyoD and β-Catenin. mDia1 associated with Akt2 and pAkt2 Ser474 specifically in MT (Fig. 4a). MyoD was also found to interact with mDia1 only during differentiation, along with Phb2 and Akt2 (Fig. 4b). Further, active β-Catenin was pulled down with mDia1 along with Phb2 (Fig. 4c). Interestingly, mDia1 also co-immunoprecipitated the transcriptional regulator Prohibitin1 (Phb1), a known partner of Phb2^61,63^, suggesting a role for this complex in gene regulation (Fig. 4d). Akt2 and Phb2 were also co-immunoprecipitated by mDia1 along with Phb1. These results indicate that mDia1 may participate in multi-protein complexes that contain differentiation-regulating proteins specifically in MT. Taken together, our findings suggest that the mDia1-Phb2 protein complex might also contain one or more of the mDia1-interacting partners pAkt2 Ser474, MyoD and active β-Catenin to regulate differentiation.

### Over-expression of mDia1 leads to repression of Myogenin, which is reversed by co-expressed Phb2

**Figure 5:**
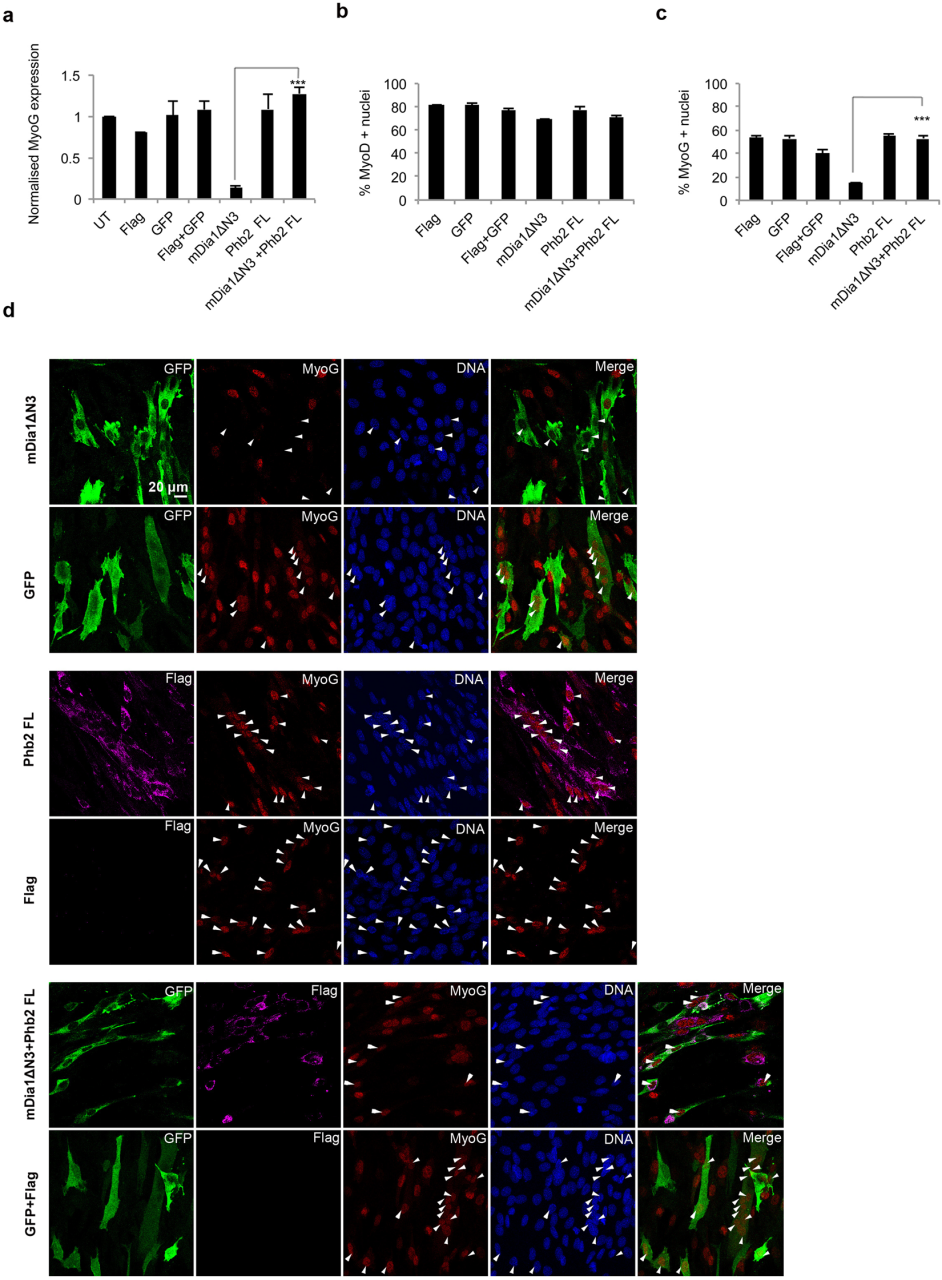
Co-expression of mDia1 and Phb2 prevents repression of endogenous MyoG and MyoD. (**a)** Overexpressed mDia1ΔN3 represses MyoG in MT, while co-expressed Phb2 reverses the repression. qRT-PCR analysis of MyoG transcripts in C2C12 transiently transfected with mDia1ΔN3 and Phb2 and shifted to DM for 36 hours. ***p < 0.001, n = 3. Bar graphs indicate normalised mRNA values. UT and GAPDH were used for normalisation of mRNA levels. Error bars represent ± s.e.m. (**b**,**c**) MyoG protein (but not MyoD) is repressed by mDia1ΔN3. Immunostaining of endogenous MyoD and MyoG in MT ectopically expressing mDia1 and Phb2. C2C12 were transfected with mDia1ΔN3 and Phb2 FL and shifted to DM for 36 hours, followed by immunostaining for Flag (Phb2), GFP (mDia1), MyoD and MyoG. Percentage of MyoD (**b**) and MyoG (**c**) positive nuclei were determined by counting at least 200 transfected cells. ***p < 0.001, n = 3. (**d**) Representative images of endogenous MyoG protein during over-expression of mDia1ΔN3 and Phb2 during differentiation. UT-Untransfected, FL-Full-length.

Association of mDia1 and Phb2 with pro-myogenic proteins prompted us to explore a role for mDia1 and Phb2 in regulating expression of the key transcriptional regulator of differentiation, MyoG. Using over-expression studies in MT (Fig. 5a) we found that while expression of flag-tagged Phb2 FL alone did not affect MyoG transcript levels, expression of GFP-tagged mDia1∆N3 alone strongly suppressed the level of MyoG transcripts to 13% of control. Notably, when Phb2 was co-expressed with mDia1∆N3, MyoG mRNA level was restored to control levels, suggesting that the interaction counteracts repression by mDia1. To further assess the effect of ectopic Phb2 and mDia1∆N3 on MyoD and MyoG protein expression in MT, we used immunofluorescence. Ectopic expression of mDia1∆N3 and Phb2 did not affect the number of MyoD+ cells (Fig. 5c). Phb2 FL expression on its own did not affect MyoG, while mDia1∆N3 expression reduced the number of MyoG+ cells to 15%. However, co-expression of Phb2 with mDia1∆N3 restored the frequency of MyoG+ cells to 53%, comparable to control (Fig. 5d,e). Thus, increasing mDia1 levels represses MyoG expression at the level of mRNA and protein, with no effect on MyoD protein levels. Co-expression of Phb2 blocks mDia1’s repressive effect on MyoG. Taken together with endogenous expression of mDia1 and Phb2 (Fig. 1i–k), the results so far suggest that mDia1 possesses an anti-myogenic activity, which may be modulated by Phb2 specifically in MT to permit efficient differentiation.

### Phb2 relieves mDia1ΔN3-mediated repression of MyoG promoter

**Figure 6:**
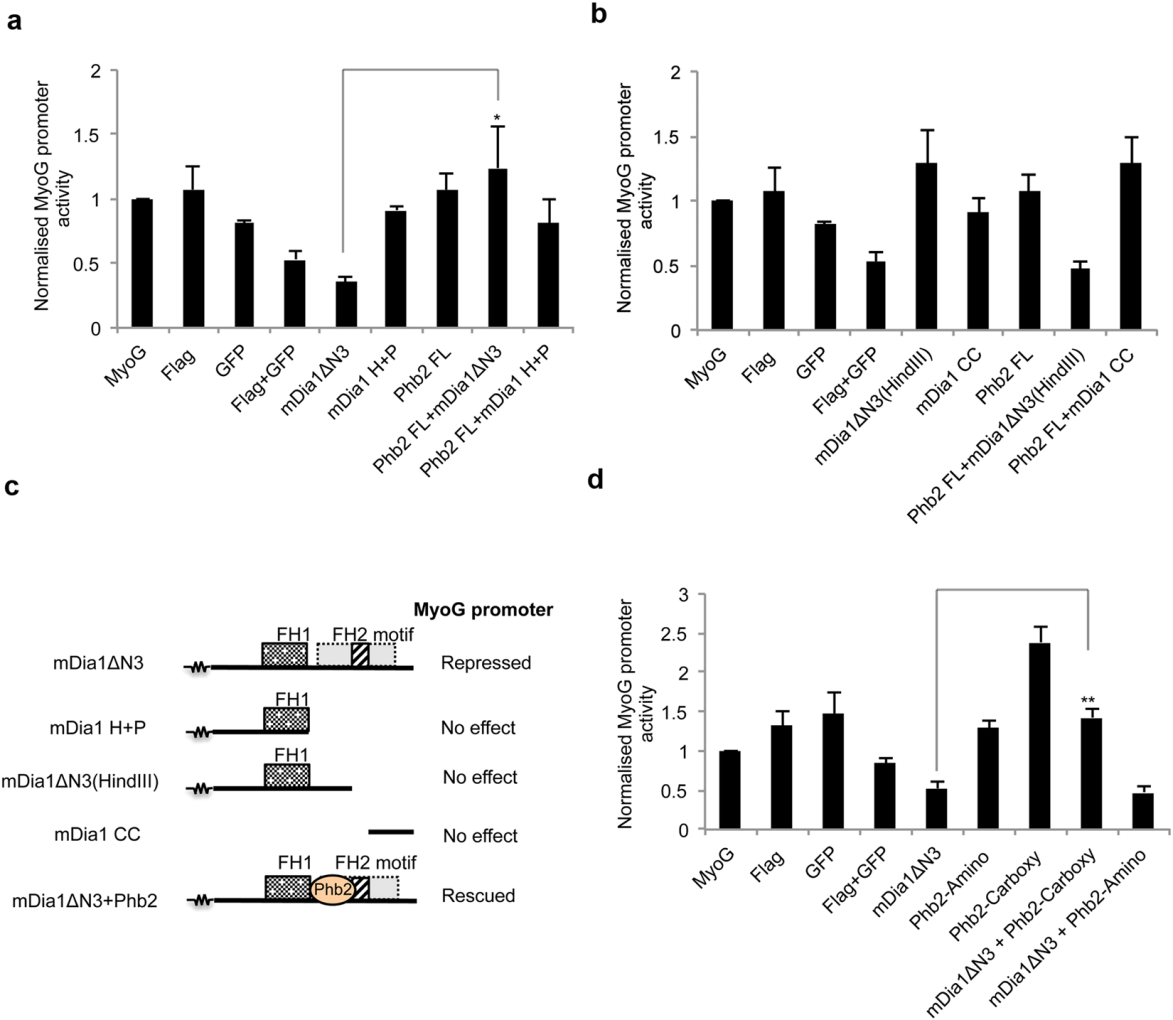
Co-expression of mDia1ΔN3 and Phb2 rescues MyoG promoter activity. C2C12 were transfected with various mDia1 and Phb2 mutants along with MyoG-promoter reporter construct and shifted to DM for 72 hours, followed by lysis and dual-luciferase assays. (**a**) Normalised MyoG promoter activity in MT transfected with mDia1ΔN3, mDia1H + P or Phb2 FL. *p < 0.05, n = 3. (**b**) Normalised MyoG promoter activity in MT transfected with mDia1ΔN3(HindIII), mDia1CC or Phb2 FL, n = 3. (**c**) Schematic illustrating FH2 motif (aa 946–1010)-mediated regulation of MyoG promoter by mDia1 mutants and Phb2. The squiggle represents the common domains not depicted. The FH2 motif is indicated by the stripped box within the dotted grey box representing the FH2 domain. (**d**) Normalised MyoG promoter activity in MT transfected with mDia1ΔN3, Phb2-Carboxy or Phb2-Amino. **p < 0.01, n = 3. For all Luciferase assays performed, Luciferase readings were normalised to Renilla Luciferase, empty pGL3 vector and basal DRR or MyoG promoter activity, to correct for background luminescence and transfection efficiency. Bar graphs represent normalised Luciferase values. Error bars represent ± s.e.m. FL-Full-length. UT-Untransfected.

To further evaluate the functional significance of mDia1 and Phb2 interaction, we studied the effect of ectopically expressed mDia1ΔN3 and Phb2 on MyoG promoter activity. MyoG promoter-luc constructs^64^ were transfected into C2C12 cells along with individual mDia1 mutants and Phb2 FL during differentiation. In MT, mDia1∆N3 individually reduced the activity of the MyoG promoter whereas Phb2 did not (Fig. 6a). As with endogenous MyoG expression, when Phb2 was co-expressed with mDia1∆N3, MyoG promoter activity returned to control levels. Co-expression of Phb2 with the non-interacting mDia1 mutant mDia1H+ P did not affect MyoG promoter activity. Thus, Phb2 interaction with mDia1∆N3 is required to rescue mDia1-mediated repression of MyoG promoter activity.

Other mDia1 mutants mDia1∆N3(HindIII) and mDia1CC, when expressed alone or with Phb2 did not affect MyoG promoter activity (Fig. 6b), as with Phb2 alone. Only mDia1 mutants that include the FH2 motif (aa 946–1010), repressed MyoG promoter activity (Fig. 6c, Table 1). Notably, Phb2’s interaction with mDia1∆N3, which includes FH2-motif, rescued the repression of MyoG promoter. Taken together, these findings indicate that mDia1∆N3-Phb2 interaction is required to rescue MyoG promoter activity and that additional FH2-motif specific mechanisms may operate to modulate the MyoG promoter.

**Table 1:** Regulation of MyoG promoter by mDia1 mutant-Phb2 interaction.

Expressed proteins	FH2 motif	MyoG promoter	Proposed mechanism of action
mDia1ΔN3	Present	Repressed	FH2 motif-mediated repression
mDia1H + P	Absent	No effect	No FH2 motif-mediated repression
mDia1ΔN3(HindIII)	Absent	No effect	No FH2 motif-mediated repression
mDia1CC	Absent	No effect	No FH2 motif-mediated repression
Phb2	Not applicable	No effect	No effect
mDia1ΔN3 + Phb2	Present in mDia1 mutant	Rescued	Phb2 recruits pro-myogenic proteins to FH2 motif

To further delineate Phb2’s rescue of MyoG promoter activity from repression by mDia1∆N3, we used the Phb2 truncation constructs. As seen earlier, mDia1∆N3 on its own repressed MyoG promoter activity (Fig. 6d). Co-expression of mDia1-interacting Phb2 mutant, Phb2-Carboxy rescued MyoG promoter activity, but Phb2-Amino, a mutant that does not interact with mDia1 did not. Consistent with the results using different mDia1 domains, only the Phb2-Carboxy mutant that included the mDia1-interacting region rescued the MyoG promoter activity, while Phb2-Amino that lacked the mDia1-interacting region did not. On its own, over-expression of the mDia1-interacting Phb2-Carboxy mutant induced MyoG promoter activity, consistent with sequestering endogenous mDia1, while the non-interacting Phb2-Amino did not. Together, these findings further emphasise a role for Phb2 in mitigating the repressive effect of mDia1 on MyoG promoter activity in MT.

**Figure 7:**
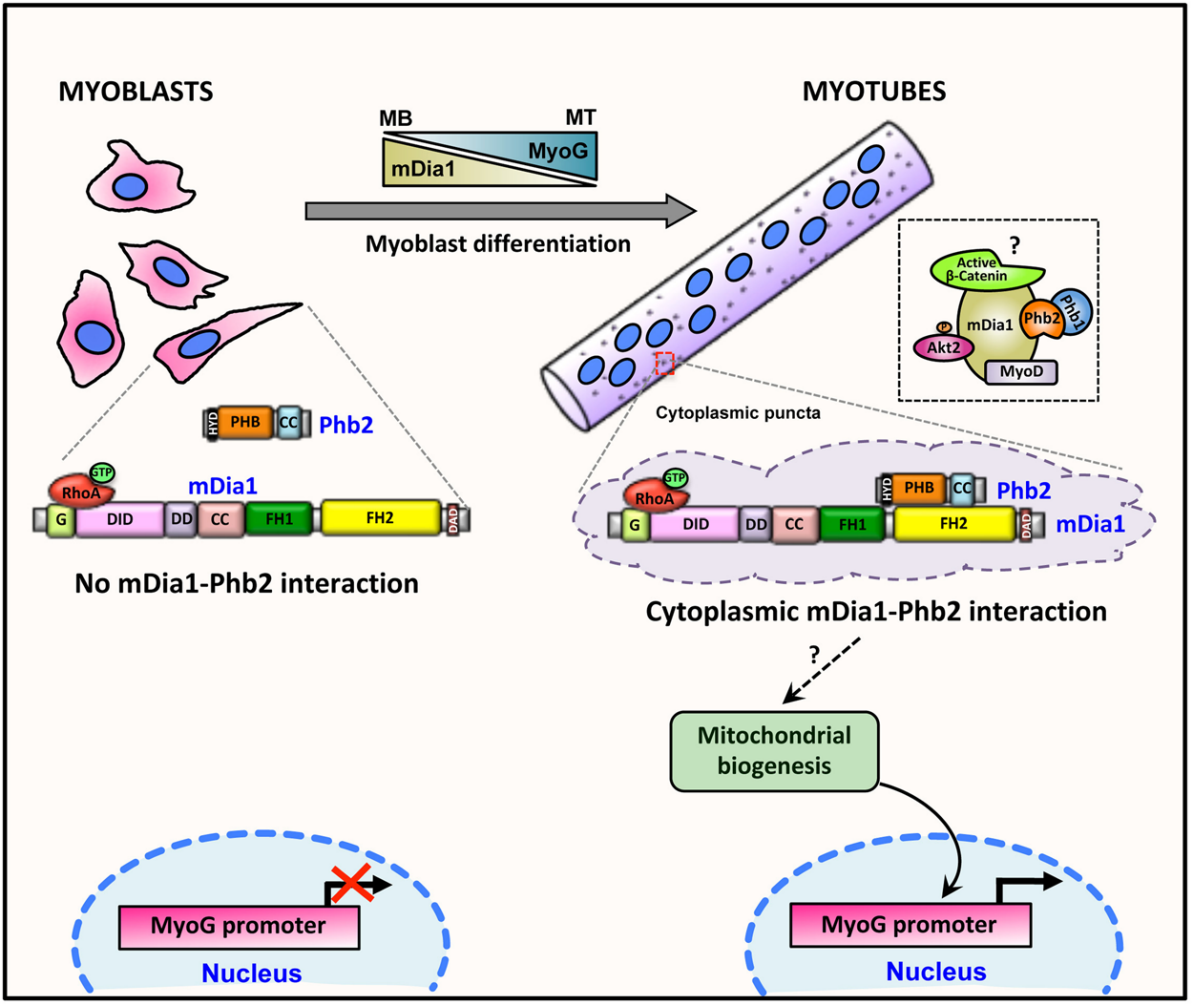
Model: Phb2 sequesters mDia1 in the cytoplasmic puncta during differentiation to promote MyoG expression. mDia1 does not interact with Phb2 in MB and its expression decreases during differentiation. In MT, Phb2 binds and sequesters mDia1 in the cytoplasmic puncta, thereby restricting its availability and anti-myogenic activity to promote MyoG expression. The mDia1-Phb2 interaction might promote mitochondrial biogenesis in MT, thereby enhancing MyoG expression and differentiation. Although we have shown that mDia1 interacts with MyoD, pAkt2 Ser474, β-Catenin and Phb1, it remains unclear whether these interactors bind the cytoplasmic mDia1-Phb2 complex to regulate MyoG expression or exist as separate mDia1-interacting pools. Black dotted box indicates a possible mDia1 complex that might regulate MyoG in MT, but needs additional studies. Red dotted box highlights a cytoplasmic puncta. Dashed arrow highlights studies that need to be investigated.

In summary, we report that mDia1 is involved in differentiation-specific interactions with multiple transcriptional regulators Phb2, MyoD, pAkt2 Ser474 and active β-Catenin, suggesting the involvement of one or more complexes of signalling molecules and TFs focused on control of MyoG expression (Fig. 7). Exogenously expressed mDia1 represses MyoG promoter activity as well as its transcript and protein. However, when bound to Phb2, mDia1 does not repress MyoG, suggesting that the differentiation-specific interaction of mDia1-Phb2 is required to block mDia1-mediated repression of MyoG. Moreover, the mDia1-Phb2 interaction localises to cytoplasmic puncta in MT, indicating that Phb2 may sequester mDia1 to regulate its anti-myogenic activity and mitigate repression of MyoG. In the context of earlier reports showing that activated RhoA represses myogenesis, our findings suggest that RhoA effector mDia1 is a mediator of this effect, and that counter-mechanisms involving Phb2 have evolved to preserve differentiation capability.

## Discussion

We report that during muscle differentiation, signalling mediated by RhoA effector mDia1 is anti-myogenic, and identify a new mDia1-interacting protein, the multi-functional Phb2 that mitigates these effects to facilitate progression of the myogenic program. We map the domains by which Phb2 and mDia1 interact. We demonstrate that mDia1 represses MyoG expression in MT, and that this repression is relieved by interaction with Phb2. We further demonstrate that mDia1 interacts with differentiation-promoting TFs MyoD, pAkt2 Ser474 and active β-Catenin in MT. We implicate mDia1 as a scaffold molecule with the potential to bind many proteins that might regulate its anti-myogenic activity and impact multiple pathways in a stage-specific manner. Finally, we propose a model wherein mDia1’s anti-myogenic activity is modulated by Phb2-mediated sequestration of mDia1 in cytoplasmic puncta in MT to promote MyoG expression and allow efficient differentiation.

Our studies place RhoA effector mDia1 as a negative regulator of MyoD function and MyoG expression during differentiation. Consistent with known repressive effects of RhoA on differentiation^15,18,20,65–67^, we report that ectopic constitutively active mDia1ΔN3 represses MyoG expression but does not alter MyoD protein levels. MyoG promoter activity, mRNA levels and protein abundance were all reduced by mDia1ΔN3, indicating that high mDia1 activity is repressive. Taken together, our findings suggest that high mDia1 activity is anti-myogenic during differentiation, and may mediate the known repressive effects of RhoA in myogenesis.

The activity of mDia1 and its isoforms needs to be tightly regulated to promote optimal function^33,68^. Reduced mDia1 expression during myotube formation supports this notion, and we provide further mechanistic evidence that mDia1’s anti-myogenic activity must be dampened in order to promote differentiation, (Fig. 1i–k). As a key regulator of actin dynamics, continued mDia1 activity would be required during myogenesis, given the importance of acto-myosin contractility in the process. However, domain-specific interactions with proteins particularly in MT may have evolved to mitigate the repressive effects of mDia1 on upstream, non-cytoskeletal functions and thereby preserve differentiation ability. Interaction of mDia1 with Phb2 was restricted to the cytoplasm in MT, as evidenced both by microscopy and IP, and suggests that Phb2 sequesters mDia1 in cytoplasmic puncta to regulate its anti-myogenic activity. Our findings are consistent with studies that report RhoA activity needs to be down-regulated prior to fusion to promote differentiation^17–20^. Thus, cytoplasmic sequestration of mDia1 by Phb2 provides a mechanism for down-regulating anti-myogenic aspects of RhoA signalling. In MB, where MyoG is not transcriptionally activated, Phb2 and mDia1 do not interact. In this context, we show that the binding of Phb2 to mDia1 specifically in MT is pro-myogenic, counteracting the anti-myogenic effects of mDia1. Our findings contrast with the anti-myogenic role earlier ascribed to Phb2^42,43^, possibly since those reports used synthetic reporters containing control elements that do not reflect the endogenous promoters, and lacked direct loss-of-function studies in MT. In addition, those studies did not report the interaction of Phb1, a known interactor for Phb2^60,69,70^, whereas we detect interactions of mDia1 with Phb2 along with Phb1, and Akt2. Our findings thus add to Phb2’s known transcriptional control function^38–40^.

The domain mapping of Phb2-mDia1 interaction reveals mechanistic avenues. Phb2 binds to aa 752–978 region of mDia1 which overlaps with the FH2 motif (aa 946–1010) involved in repressing MyoG promoter activity. Conceivably, binding of Phb2 to aa 752–978 region may block mDia1-mediated negative regulation of MyoG by recruiting pro-myogenic regulators to the FH2 motif, promoting MyoG expression. In support of this hypothesis, we find that mDia1 binds pro-myogenic factors MyoD, Akt2, and β-Catenin in MT and that the associated Akt2 is active (pSer474)^71^. MyoD is required for MyoG transcriptional induction^72,73^, Akt2 induces MyoG expression^74^, while β-Catenin promotes MyoD expression and function^75–78^. Interestingly, Insulin and Wnt pathways cooperate to promote myogenesis^79^. Currently, it is unclear which of these additional mDia1-associated proteins may promote rescue of MyoG expression. Taken together, our data suggests that domain-specific interactions of mDia1 with Phb2, MyoD, Akt2, and β-Catenin might regulate MyoG expression.

mDia1 thus emerges as a node for several protein interactions, which in turn might mitigate its anti-myogenic functions. We speculate that upon binding Phb2, mDia1 may act as a scaffold to form a complex with MyoD, pAkt2 Ser474, β-Catenin or Phb1 to promote or sustain differentiation. Although Phb1 has not been reported to regulate myogenesis, it represses E2F-dependent transcription^80–82^ and might promote irreversible cycle exit during differentiation. Taken together, these findings suggest the formation of a pro-myogenic complex restricting stage-specific mDia1 functions. However, we cannot currently distinguish whether pAkt2 Ser474, MyoD, Phb1 and active β-Catenin simultaneously associate with the mDia1-Phb2 complex or exist as different mDia1-bound complexes.

mDia1 plays a stage-specific role in regulating muscle-specific gene expression in MB and MT owing to its stage-specific interactions.While our previous studies showed that ectopic mDia1 repressed MyoD protein levels in MB^33^, it did not do so in MT (this study). It is possible that post-transcriptional modifications (PTMs) of either Phb2 or mDia1 may act to restrict this interaction to MT despite expression of both proteins in MB. However, a restricted survey did not reveal altered PTMs of Phb2 (Supplementary Fig. S4) in a stage-specific manner, and this issue requires a more comprehensive analysis. Further, the substantial impact of mDia1 on MyoG transcription and the rescue by Phb2 (Figs 5 and 6) despite low cytoplasmic interaction in punctae may reflect highly dynamic interactions that are not sufficiently visualised in fixed cells, and may be resolved by live-imaging.

Additionally, LC-MS/MS analysis of mDia1-interacting proteins revealed that mDia1 may act as a scaffold for both common and stage-specific partners which might govern its functions in MB and MT. mDia1 associates with cytoskeletal regulators in both MB and MT, suggesting that its indispensable role in cytoskeletal dynamics is preserved. We report that mDia1 associates with actin binding proteins (ABP) cofilin1^83^ and profilin, a known interactor^24^ in both MB and MT. mDia1 binds a plethora of metabolic, mitochondrial, and proteasomal proteins only in MT. Mitochondria are potential regulators of myogenesis^84^ and proteasomes regulate mitochondrial health^85,86^. Thus, mDia1 may modulate a network of mitochondrial, metabolic and proteasomal functions to regulate myogenesis.

The direct transciptional mechanism through which the mDia1-Phb2 interaction promotes MyoG expression remains to be elucidated. SRF and TCF, TFs reported to act downstream mDia1^33^, do not regulate MyoG expression, although SRF but not TCF activity is induced by the mDia1-Phb2 interaction (Supplementary Fig. S7). Using the interactome of mDia1 as a clue, it is tempting to speculate that mitochondrial biogenesis maybe involved, given the association of mDia1 with a number of mitochondrial proteins. Mitochondrial biogenesis has been reported to promote MyoG expression and differentiation^84,87–92^, and our data are consistent with a possible mitochondrial involvement^87^. Our finding that MyoG regulation by mDia1 is MyoD-independent also suggests an indirect mechanism. Additionally, both mDia1 and Phb2 have been reported to have mitochondria-specific roles^60,93–95^. Taken together, we propose that the mDia1-Phb2 interaction might promote mitochondrial biogenesis to induce MyoG expression and differentiation in MT.

In conclusion, we show that mDia1 has stage-specific roles in MB and MT and that these roles are modulated by newly-identified stage-specific interacting proteins. We identified Phb2 as one such mDia1-interacting protein in MT that partially sequesters mDia1 in cytoplasmic punctae, and modulates its repressive effect on MyoG expression. Prior to differentiation, mDia1 does not interact with Phb2, but during MT formation, the mDia1-Phb2 interaction together with other interacting proteins might maintain a moderate level of RhoA signaling, that sustains MyoG expression and permits differentiation.

## Methods

### Yeast two-hybrid screen

*Saccharomyces cerevisiae* strain PJ69-4A was co transformed with mDia1ΔN3-BD (pGBKT7 –GAL4 Binding domain vector) and Matchmaker 7 day old mouse embryonic cDNA library cloned in pACT2 (AD-GAL4 Activation domain vector) (Clonetech). Transformants were screened for *ADE2* and *LacZ* reporter expression on amino acid dropout selection plates −TLA (−Trp/−Leu/−Ade) lacking Trp (tryptophan), Leu (leucine) and Adenine (Ade) and −TL + X-Gal (−Trp/−Leu/+X-Gal) respectively. *ADE2* expression was indicated by growth wherease blue pigmented colonies indicated *LacZ* expression. PJ69-4A co-transformed with *Drosophila* Trithorax and GAGA factor served as positive control and co-transformation with empty pGBKT7 and pACT2 vectors served as negative control. Clones positive for expression of both reporters were selected for hybrid-reconstitution assays. For this assay, PJ69-4A was co-transformed with pACT2 vector from the positive clone and mDia1ΔN3-BD or empty pGBKT7, followed by plating on −TLA and −TL + X-Gal plates. Four colonies for each co-transformation per clone were screened three times serially on reporter plates. The identity of the positive clones was derived by sequencing the pACT2 plasmid from positive yeast clones followed by NCBI-nucleotide BLAST analysis against mouse genomic Reference RNA (Ref seq_RNA) database.

### Cell culture

Mouse C2C12 subclone A2 MB^96^ were maintained under proliferative condtions using growth medium (GM; DMEM + 20% FBS) and differentiated into MT in differentiation medium (DM; DMEM + 2% Horse serum). HEK293T cells were cultured in DMEM + 10% FBS. All media were supplemented 100 units/ml Penicillin, 100 μg/ml Streptomycin (Cat. no. 15140-163, Thermo scientific) and 2 mM Glutamax (Cat. no. 35050-079, Thermo Scientific).

### Transfections

C2C12 MB or HEK293T were transfected using Lipofectamine LTX (Cat. No. - 15338-100, Thermo scientific) as per manufacturer’s instructions. For C2C12, GM was replaced 12 hours post transfection by DM, followed by processing for immunostaining, RNA extraction or dual Luciferase assay. Normalised DNA amounts were used for transfection to get similar expression levels of all mutants used in the dual luciferase assay. For normalising expression of Phb2-Carboxy and Phb2-Amino in dual luciferase assay, transfections were performed with 10 times lesser Phb2-Carboxy DNA than that used for Phb2-Amino. Transfected HEK293T cells were used for western blot analysis or immunoprecipitation. Transfection efficiency was 60–70% for C2C12 and 90% for HEK293T. For siRNA studies, proliferating MB were transfected for 48 hours with siGenome SMART pool siRNA (mDia1 - Cat. no. M-064854-02-0050, Phb2 - Cat. No. M-040938-01-0005, and scrambled (SCR) control - Cat. No. D-001206-14-20) from Dharmacon using RNAiMax (Cat. No. -13778-150, Invitrogen), followed by immunostaining to test specificity of mDia1 and Phb2 antibodies.

### Plasmids and cloning

Expression plasmids for GFP-tagged mouse mDia1, mDia1FL, mDia1ΔN3, mDia1F2, mDia1ΔN3(HindIII), mDia1H + P and mDia1CC were gifts from S Narumiya (Watanabe *et al*. 1999). mDia1ΔN3-BD was generated by directional cloning in pGBKT7 (GAL4 binding domain vector-Clonetech) whereas Flag-tagged mouse Phb2 expression plasmids Phb2-Y2H (89–299aa), Phb2-Amino (89–180aa), Phb2-Central (140–244 aa), Phb2-Carboxy (180–299 aa) and Phb2 120–232 (120–232 aa) were generated using directional cloning in pCMV2B. Flag-tagged mouse Phb2 FL was obtained from Origene, MyoG prom-pGL3 was a gift from Eric Olson’s lab^64^, TCF reporters Super 8X TOP-flash (TCF site)/FOP-flash (mutated TCF site)^97^, and 3DA.luc^30^ were gifts from R.T. Moon, and R. Treisman respectively. pRLSV40 Renilla Luciferase plasmid and pBluescript KS were obtained from Addgene.

### RNA isolation and analysis

RNA was extracted from transfected MB differentiated for 36 hours using Trizol (Cat No. 15596-026, Thermo Scientific) as per manufacturer’s instructions. cDNA was synthesised using Superscript III (Cat No. 18080-044, Thermo Scientific), amplified by qRT-PCR using Maxima SYBR Green 2X PCR master mix (Cat No.K0222, Fermentas) and analysed in triplicates on a ABI 7900HT thermal cycler (Applied Biosystems). Amplicons were verified by sequencing and dissociation curves. Relative level of endogenous MyoG mRNA in the transfected samples was calculated with respect to untransfected control after normalising to corresponding GAPDH levels in the transfected samples. Fold change between samples was calculated using [2^(−ΔΔCt)^] method. Primers: GAPDH 5′-AAGGCCGGGGCCCACTTGAA-3′, 5′-AGCAGTTGGTGGTGCAGGATGC-3′; MyoG 5′-CAACCAGCGGCTGCCTAAAGTGG 3′, 5′-GCATTCACTGGGCACCATGGGC -3′.

### Immunostaining

Proliferating MB, MT differentiated for 72 h (D72), or transfected MT differentiated for 36 hours were fixed with 4% paraformaldehyde, and incubated with primary and secondary antibodies post permeabilisation. DNA was stained with DAPI (1 μg/ml) prior to mounting in Fluormount (Cat No. 0100-01, Southern Biotech). Confocal images were acquired on a confocal laser scanning microscope (Leica TCS SP8, Germany) using HC PL APO CS2 40X/1.3 Oil immersion objective at Zoom 1.28 for over-expression studies and HC PL APO CS2 63X/1.4 Oil immersion objective at Zoom 3 for mDia1-Phb2 colocalisation studies. For assessing specificity of mDia1 and Phb2 antibody staining in knockdown MB, Fiji (ImageJ) was used to calculate the fluorescent intensity of more than 100 cells per sample, using the formula: Corrected mean intensity = Total intensity of signal − (Area of signal × Mean background signal). Antibodies used are listed in Supplementary Table S6.

### Immunoprecipitation assays

Cells were lysed in modified RIPA buffer (50 mM Tris-HCl pH 7.4, 150 mM NaCl, 1% NP40, 0.25% Sodium deoxycholate and 1 mM EDTA) or mDia1 IP buffer (10 mM Tris-HCl pH 7.5, 150 mM NaCl, 1 mM EDTA, 1 mM EGTA, 10% Sucrose and 1% TX100) containing 1X protease and phosphatase inhibitors for 0.5 or 2 h respectively at 4 °C, and cleared by centifugation at 13,000 rpm at 4 °C for 20 min. Prior to IP, lysates containing equal protein were pre-cleared for 1 hour at 4 °C, then incubated with 3 μg primary antibody against flag, mDia1 or Phb2 for 12 hours at 4 °C, followed by addition of Protein A or G agarose beads (Santa Cruz) for 8 hours at 4 °C. Immunoprecipitates were washed with cold PBS + 0.5% Triton-X-100 and eluted in 2X Laemmli sample buffer.

### Cytoplasmic and nuclear fractionation

D72 MT were trypsinised and resuspended in 10 times the pellet size volume of mDia1 IP buffer containing 0.2% TX100, 1X protease inhibitors and phosphatase inhibitors. Samples were incubated on ice for 10 min, vortexed gently for 15 sec followed by two serial centrifugations at 4 °C for 15 min at 800 g to collect cytoplasmic fraction. The nuclear pellet was then washed with F2 buffer without detergent (20 mM Tris-HCl pH 7.6, 0.1 mM EDTA and 2 mM MgCl_2_) and lysed in mDia1 IP buffer containing 1X protease inhibitors and phosphatase inhibitors for 2 hours at 4 °C. Nuclear fractions were collected by centrifugation at 13,000 rpm for 20 min at 4 °C. Cytoplasmic and nuclear fractions were used for immunoprecipitation studies.

### Western blot analysis

Equal volume of IP product, 25–40 μg of whole cell lysates prepared in 2X SDS lysis buffer (100 mM Tris-HCl pH 6.8, 4% SDS, 10% β-mercaptoethanol and 10 mM EDTA) or cellular fractions were separated by SDS-PAGE followed by transfer to Polyvinylidene Difluoride (PVDF) membrane (Cat. no. 162-0177, Biorad). Immunoblot was incubated with primary and HRP-conjugated secondary antibodies and chemiluminescent signal was detected by ImageQuant (Amersham) or ChemDoc (Syngene) using ECL detection reagent (Amersham).

### Dual-Luciferase assays

MB were transfected with pRLSV40 Renilla Luciferase (Addgene), MyoG-promoter/3DA.luc/TOP-flash/FOP-flash/empty pGL3 Luciferase reporter constructs, mDia1ΔN3/mDia1H + P, Phb2 FL/Phb2-Carboxy/Phb2-Amino, empty pEGFPC1 or empty pCMV2B constructs and differentiated for 72 hours. pBluescript KS (pBSKS) was used to ensure equal DNA amount during transfection. Reporter expression was assayed using Dual-Luciferase kit (Cat. no. E1910, Promega). Luciferase assay reagent LARII was added to cell lysates to record firefly Luciferase activity in a TD-20/20 luminometer (Turner Designs) followed by addition of Stop and Glow to record Renilla Luciferase activity. Luciferase readings were expressed as relative light units (RLU) normalised to Renilla Luciferase for transfection and pGL3 for basal Luciferase activity.

### Mass spectrometric analysis

mDia1 immunoprecipitates from GM and D72 cultures were resolved on NuPAGE 4–12% Bis-Tris pre-cast gels (Invitrogen) followed by staining with Coomassie brilliant blue R250. Each lane was cut into smaller pieces, in-gel digested, desalted and enriched for Liquid chromatography tandem mass spectrometry (LC-MS/MS) analysis as described^98,99^. Briefly, eluted peptides from desalting tips were resuspended in 2% (v/v) formic acid and sonicated for 5 min. Samples were analysed on Q Exactive Hybrid Quadrupole-Orbitrap Mass spectrometer (Thermo Scientific) coupled to a nanoflow LC system (Easy nLC II, Thermoscientific). Peptide fractions were loaded onto a BioBasic C18 PicoFrit 15 μm nanocapillary reverse phase HPLC column (75 μm × 10 cm; New Objective, MA, USA) and separated using a 60 min linear gradient of the organic mobile phase [5% Acetonitrile (ACN) containing 0.1% formic acid and 95% ACN containing 0.1% formic acid], at a flow rate of 400 nl min^−1^. Protein/peptides were identified by searching against Swissprot amino acid sequence database of *Mus musculus* (release March 2016 with 16790 entries) and a database of known contaminants using MaxQuant software (Version 1.3.0.5)^100^. MaxQuant uses a decoy version of the specified Swissprot database to adjust the false discovery rate for proteins and peptides below 1%. The search was set up for tryptic peptides with minimum peptide length of seven aa, including constant modification of cysteine by carbamidomethylation, minimum two peptide identification and label-free quantitation (LFQ). LFQ ratio for individual proteins was calculated by LFQ in mDia1 IP/LFQ in IgG. Proteins that had LFQ ratio of 2 or greater were selected for further analysis. Three independent biological samples of MB and MT were processed for mDia1 IP-LC-MS/MS and only those proteins that were detected with significance in all three runs were selected for further analysis. Gene ontology analysis was performed using REVIGO http://revigo.irb.hr.

## Supplementary information

Supplementary Information

## Data Availability

The mass spectrometry proteomics data generated during this study have been deposited to the ProteomeXchange Consortium (http://proteomecentral.proteomexchange.org) via the PRIDE partner repository^101^ with the dataset identifier PXD012257.
