# Supplementary material for: Cytoplasmic sequestration of the RhoA effector mDiaphanous1 by Prohibitin2 promotes muscle differentiation
Source: Sci Rep. 2019 Jun 5;9:8302. doi: 10.1038/s41598-019-44749-4 (PMC6549159; doi:10.1038/s41598-019-44749-4)

Cytoplasmic sequestration of the RhoA effector mDiaphanous1 by Prohibitin2 promotes muscle differentiation

Amena Saleh[1,2,3], Gunasekaran Subramaniam[2,4], Swasti Raychaudhuri[2] & Jyotsna Dhawan[1,2*]

[1]Institute for Stem Cell Biology & Regenerative Medicine, Bangalore, Karnataka- 560065, India

[2] Council of Scientific & Industrial Research -Centre for Cellular & Molecular Biology, Hyderabad, Telangana- 500007, India

[3] Manipal Academy of Higher Education, Manipal, Karnataka- 576104, India

[4] Department of Physiology, Anatomy and Genetics, University of Oxford, Oxford OX1 3PT, UK

*Corresponding author

E-mail: jdhawan@ccmb.res.in, jdhawan@instem.res.in

Supplementary information

Supplementary Figure S1. Putative mDia1-interacting proteins identified in the yeast two-hybrid screen. Yeast two-hybrid screen to identify novel mDia1-interacting proteins. A GAL4 hybrid reconstitution assay with putative mDia1 interactors was performed to study the induction of reporters *ADE2* and *LacZ* on -Trp/-Leu/-Ade and -Trp/-Leu+X-Gal respectively. PJ69-4A was co transformed with interacting protein plasmid and mDia1ΔN3-BD (positive reconstitution) or empty BD vector (negative reconstitution). Four colonies per per reconstitution assay (positive and negative) were screened on selection plates. PJ69-4A co-transformed with *bona fide* interacting proteins *Drosophila* Batman-AD and GAGA factor-BD served as a positive control "P" for reporter expression and PJ69-4A co-transformed with empty pACT2 and pGBKT7 vectors served as a negative control "N". Induction of *ADE2* reporter is indicated by growth and induction of *LacZ* expression is indicated by blue colour in the colonies on the selection plates. Seven library clones that induced the reporter expression were finally selected from the screen as mDia1-interacting proteins of interest. (**a**) Profilin1 (Pfn1), known interactor for mDia1 (**b**) Cadherin11 (Cdh11) (**c**) Niemann Pick Type C2 (Npc2) (**d**) Leukocyte receptor cluster (LRC) member 8 (Leng8) (**e**) Growth factor receptor bound protein 2 (Grb2) (**f**) Protein-kinase, interferon-inducible double stranded RNA-dependent inhibitor, repressor of (p58 repressor) (Prkrir) (**g**) Cytochrome c1 (Cyc1) were identified as mDia1-interacting proteins. Trp-Tryptophan, Leu-Leucine, Ade-Adenine.

Supplementary Figure S2. Gene Ontology of mDia1-interacting proteins identified in MB and D72 MT by mDia1 IP-LC-MS/MS analysis. Gene ontology analysis of mDia1-interacting proteins identified by mDia1 IP-LC-MS/MS analysis in myoblasts (MB) and myotubes (MT) lysates was performed using REVIGO based on associated biological processes and cellular components. Gene ontology of proteins that bind mDia1 in both MB and MT based on (**a**) biological process and (**b**) cellular components. Gene ontology of mDia1-interacting proteins in MB based on (**c**) biological process and (**d**) cellular components. Gene ontology of the proteins that bind mDia1 in MT based on (**e**) biological process and (**f**) cellular components.

Supplementary Figure S3. Protein association networks for mDia1-interacting proteins in MB and D72 MT generated by STRING. STRING analysis of mDia1-interacting proteins in (**a**) both MB and MT, (**b**) MB and (**c**) MT. Highlighted clusters- Proteasomal proteins (Red),

metabolic enzymes (Blue), mitochondrial proteins (Black).

Supplementary Figure S4. Phosphorylation of Phb2 at Tyr128 and Tyr248 residues remains unchanged during differentiation. Lysates from proliferating myoblasts (GM) and MT (D72) were collected and subjected to western blot analysis using antibodies against phosphorylated Phb2. Western blots indicate the protein levels of Phb2 phosphorylated at (**a**) Tyr128 and (**b**) Tyr248. GAPDH and total Phb2 levels serve as controls to normalise the expression of phosphorylated Phb2 (pPhb2). (**c, d**) Uncropped versions of the blots shown in a and b respectively. Arrow marks indicate the protein band corresponding to Phb2 phoshorylated at Tyr248.

Supplementary Figure S5. Validation of the antibodies used for colocalisation analysis. (**a**) Immunostaining of mDia1 and Phb2 in mDia1 or Phb2 knockdown myoblasts respectively. MB were transfected with control scrambled (SCR), mDia1 (si_mDia1) or Phb2 (si_Phb2) siRNA pools and cultured in growth medium for 48 hours, followed by immunostaining with anti-mDia1 and anti-Phb2 antibodies used in colocalisation analysis. Arrows mark myoblasts showing knockdown at the level of protein abundance. Box plots represent the corrected mean intensity of (**b**) mDia1 and (**c**) Phb2 in immunostained myoblasts represented in Fig. S4a. ***$p<0.001$, n=3. (**d**) A representative western blot showing the knockdown of mDia1 and Phb2 in myoblasts. Densitometric quantification of (**e**) mDia1 and (**f**) Phb2 knockdown is shown, ***$p<0.001$, n=3. (**g**) IP of mDia1 in D72 MT by anti-Phb2 antibody used for colocalisation analysis. IP product was loaded on different gels, the blot was cut and processed in parallel under the same conditions of detection for mDia1 and Phb2.

Supplementary Figure S6. Representation of uncropped blots. (**a**) Representative full-length uncropped GFP blot of the blots shown in Fig. 1d. (**b, c, d**) Uncropped blots of the blots shown in Fig. 4b, c, d respectively.

Supplementary Figure S7. SRF but not TCF activity is regulated by the mDia1-Phb2 interaction. Dual luciferase assays were performed using lysates from C2C12 transfected with TCF reporters TOP-flash/FOP flash or SRF reporter 3DA.luc, together with various mDia1 mutants and/or Phb2 FL and shifted to DM for 72 hours. (**a**) Normalised TCF activity in MT

transfected with mDia1ΔN3, mDia1H+P or Phb2 FL, n=3. The ratio of TOP/FOP activity determines the normalised TCF activity. (**b**) Normalised SRF activity in MT transfected with mDia1ΔN3, mDia1H+P or Phb2 FL, *$p<0.05$, n=3.

Supplementary Table S1. mDia1-interacting proteins identified in Y2H.

S.no	Name	Symbol	Yeast clone	Gene ID
1	195B	Profilin 1	Pfn1	18643
2	194A	Prohibitin2	Phb2	12034
3	169A, 173A	Growth factor receptor bound protein 2	Grb2	14784
4	190D, 214A	Niemann Pick type C2	Npc2	67963
5	175A, 291A, 292A, 327A	Cadherin 11	Cdh11	12552
6	176A	Leukocyte receptor cluster (LRC) member 8	Leng8	232798
7	211A	Protein-kinase, interferon-inducible double stranded RNA-dependent inhibitor, repressor of (P58 repressor)	Prkrir	72981
8	191A	Cytochrome c-1	Cyc1	66445

Supplementary Table S2. mDia1-interacting proteins commonly identified in MB and MT by LC-MS/MS.

S.no.	Name	Symbol	UniProt ID
1	Protein diaphanous homolog 1	Diaph1	O08808
2	Annexin A1	Anxa1	P10107
3	Annexin A6	Anxa6	P14824

4	Cofilin-1	Cfl1	P18760
5	Elongation factor 2	Eef2	P58252;O08810
6	Galectin-1	Lgals1	P16045
7	Profilin-1	Pfn1	P62962;CON__P02584
8	Pyruvate kinase isozymes M1/M2	Pkm2	P52480;P53657
9	Peptidyl-prolyl cis-trans isomerase A	Ppia	P17742
10	GTP-binding nuclear protein Ran	Ran	P62827;Q61820
11	Serpin H1	Serpinh1	P19324
12	14-3-3 protein zeta/delta	Ywhaz	P63101;P62259;O70456
13	ATP synthase subunit beta, mitochondrial	Atp5b	P56480

Supplementary Table S3. mDia1-interacting proteins identified specifically in MB by LC-MS/MS.

S.no.	Name	Symbol	UniProt ID
1	Actin, aortic smooth muscle;Actin, gamma-enteric smooth muscle	Acta2;Actg2	P62737;P63268
2	Heat shock protein HSP 90-alpha	Hsp90aa1	P07901
3	Heat shock protein HSP 90-beta	Hsp90ab1	P11499
4	Endoplasmin	Hsp90b1	P08113
5	Importin subunit beta-1	Kpnb1	P70168
6	Protein disulfide-isomerase A3	Pdia3	P27773
7	Peroxiredoxin-1	Prdx1	P35700
8	60S ribosomal protein L31	Rpl31	P62900
9	40S ribosomal protein S12	Rps12	P63323

10	Transgelin	Tagln	P37804
11	Transgelin-2	Tagln2	Q9WVA4;Q9R1Q8

Supplementary Table S4. mDia1-interacting proteins identified specifically in MT by LC-MS/MS.

S.no.	Name	Symbol	UniProt ID
1	3-ketoacyl-CoA thiolase, mitochondrial	Acaa2	Q8BWT1
2	Short-chain specific acyl-CoA dehydrogenase, mitochondrial	Acads	Q07417
3	Acetyl-CoA acetyltransferase, mitochondrial	Acat1	Q8QZT1
4	Adenylate kinase isoenzyme 1	Ak1	Q9R0Y5
5	Aldose reductase	Akr1b1	P45376
6	Fructose-bisphosphate aldolase A	Aldoa	P05064
7	Annexin A2	Anxa2	P07356
8	Annexin A5	Anxa5	P48036
9	ADP-ribosylation factor 3;ADP-ribosylation factor 1;ADP-ribosylation factor 2	Arf3;Arf1; Arf2	P61205;P84078;Q8BSL7
10	Sarcoplasmic/endoplasmic reticulum calcium ATPase 1	Atp2a1	Q8R429
11	ATP synthase subunit b, mitochondrial	Atp5f1	Q9CQQ7
12	ATP synthase subunit d, mitochondrial	Atp5h	Q9DCX2
13	ATP synthase subunit e, mitochondrial	Atp5i	Q06185
14	Carbonic anhydrase 3	Ca3	P16015
15	Creatine kinase B-type	Ckb	Q04447
16	Creatine kinase M-type	Ckm	P07310
17	Calponin-3	Cnn3	Q9DAW9

18	NADH-cytochrome b5 reductase 3;NADH-cytochrome b5 reductase 3 membrane-bound form;NADH-cytochrome b5 reductase 3 soluble form	Cyb5r3	Q9DCN2
19	Cytochrome c1, heme protein, mitochondrial	Cyc1	Q9D0M3
20	Destrin	Dstn	Q9R0P5
21	Eukaryotic translation initiation factor 4H	Eif4h	Q9WUK2
22	Alpha-enolase	Eno1	P17182
23	Beta-enolase	Eno3	P21550
24	S-formylglutathione hydrolase	Esd	Q9R0P3
25	Fatty acid synthase;[Acyl-carrier-protein] S-acetyltransferase;[Acyl-carrier-protein] S-malonyltransferase;3-oxoacyl-[acyl-carrier-protein] synthase;3-oxoacyl-[acyl-carrier-protein] reductase;3-hydroxypalmitoyl-[acyl-carrier-protein] dehydratase;Enoyl-[acyl-carrier-protein] reductase;Oleoyl-[acyl-carrier-protein] hydrolase	Fasn	P19096
26	Glyceraldehyde-3-phosphate dehydrogenase	Gapdh	P16858
27	Rab GDP dissociation inhibitor beta	Gdi2	Q61598
28	Lactoylglutathione lyase	Glo1	Q9CPU0
29	Aspartate aminotransferase, cytoplasmic	Got1	P05201
30	Aspartate aminotransferase, mitochondrial	Got2	P05202
31	Histone H1.1	Hist1h1a	P43275
32	10 kDa heat shock protein, mitochondrial	Hspe1	Q64433
33	Heat shock protein beta-1	Hspb1	P14602
34	Isocitrate dehydrogenase [NAD] subunit alpha, mitochondrial	Idh3a	Q9D6R2
35	Ras GTPase-activating-like protein IQGAP1	Iqgap1	Q9JKF1

36	Leucine-tRNA ligase, cytoplasmic	Lars	Q8BMJ2
37	L-lactate dehydrogenase A chain	Ldha	P06151
38	Galectin-3	Lgals3	P16110
39	LIM domain-containing protein 2	Limd2	Q8BGB5
40	Leucine-rich PPR motif-containing protein, mitochondrial	Lrpprc	Q6PB66
41	Calcium uniporter protein, mitochondrial	Mcu	Q3UMR5
42	Malate dehydrogenase, cytoplasmic	Mdh1	P14152
43	Malate dehydrogenase, mitochondrial	Mdh2	P08249
44	Mitochondrial carrier homolog 2	Mtch2	Q791V5
45	Cytochrome c oxidase subunit 2	Mtco2	P00405
46	Nascent polypeptide-associated complex subunit alpha, muscle-specific form;Nascent polypeptide-associated complex subunit alpha	Naca	P70670;Q60817
47	NADH dehydrogenase [ubiquinone] iron-sulfur protein 3, mitochondrial	Ndufs3	Q9DCT2
48	Platelet-activating factor acetylhydrolase IB subunit beta	Pafah1b2	Q61206
49	Poly(rC)-binding protein 1	Pcbp1	P60335
50	Protein disulfide-isomerase A6	Pdia6	Q922R8
51	PDZ and LIM domain protein 1	Pdlim1	O70400
52	Phosphoglycerate mutase 1	Pgam1	Q9DBJ1
53	Phosphoglycerate kinase 1	Pgk1	P09411
54	Prohibitin	Phb	P67778
55	Prohibitin-2	Phb2	O35129
56	Peroxiredoxin-2	Prdx2	Q61171

57	Peroxiredoxin-4	Prdx4	O08807
58	Peroxiredoxin-6	Prdx6	O08709
59	Phosphoserine aminotransferase	Psat1	Q99K85
60	Proteasome subunit alpha type-1	Psma1	Q9R1P4
61	Proteasome subunit alpha type-4	Psma4	Q9R1P0
62	Proteasome subunit alpha type-5	Psma5	Q9Z2U1
63	Proteasome subunit alpha type-6	Psma6	Q9QUM9
64	Proteasome subunit alpha type-7;Proteasome subunit alpha type-7-like	Psma7;Psma8	Q9Z2U0;Q9CWH6
65	Proteasome subunit beta type-1	Psmb1	O09061
66	Proteasome subunit beta type-3	Psmb3	Q9R1P1
67	Proteasome subunit beta type-5	Psmb5	O55234
68	26S protease regulatory subunit 10B	Psmc6	P62334
69	26S proteasome non-ATPase regulatory subunit 13	Psmd13	Q9WVJ2
70	26S proteasome non-ATPase regulatory subunit 2	Psmd2	Q8VDM4
71	26S proteasome non-ATPase regulatory subunit 8	Psmd8	Q9CX56
72	Ras-related protein Rab-7a	Rab7a	P51150
73	60S ribosomal protein L21	Rpl21	O09167
74	60S ribosomal protein L3	Rpl3	P27659
75	60S ribosomal protein L4	Rpl4	Q9D8E6
76	60S acidic ribosomal protein P0	Rplp0	P14869
77	60S acidic ribosomal protein P2	Rplp2	P99027
78	40S ribosomal protein S15	Rps15	P62843
79	40S ribosomal protein SA	Rpsa	P14206

80	Ribosome-binding protein 1	Rrbp1	Q99PL5
81	Ras suppressor protein 1	Rsu1	Q01730
82	Reticulon-4	Rtn4	Q99P72
83	Protein S100-A4	S100a4	P07091
84	Succinate dehydrogenase [ubiquinone] iron-sulfur subunit, mitochondrial	Sdhb	Q9CQA3
85	Serpin B6	Serpinb6	Q60854
86	Rho GDP-dissociation inhibitor 1	Arhgdia	Q99PT1
87	Protein SET	Set	Q9EQU5
88	Superoxide dismutase [Cu-Zn]	Sod1	P08228
89	Serine/arginine-rich splicing factor 2	Srsf2	Q62093
90	Transaldolase	Taldo1	Q93092
91	Talin-1	Tln1	P26039
92	Triosephosphate isomerase	Tpi1	P17751
93	Translin	Tsn	Q62348
94	Tubulin alpha-4A chain	Tuba4a	P68368
95	Thioredoxin-like protein 1	Txnl1	Q8CDN6
96	Ubiquitin-conjugating enzyme E2 variant 1;Ubiquitin-conjugating enzyme E2 variant 2	Ube2v1;Ube2v2	Q9CZY3;Q9D2M8
97	Cytochrome b-c1 complex subunit 2, mitochondrial	Uqcrc2	Q9DB77
98	Vesicle-associated membrane protein-associated protein B	Vapb	Q9QY76
99	Vinculin	Vcl	Q64727
100	Voltage-dependent anion-selective channel protein 1	Vdac1	Q60932

101	Voltage-dependent anion-selective channel protein 2	Vdac2	Q60930
102	Voltage-dependent anion-selective channel protein 3	Vdac3	Q60931
103	14-3-3 protein beta/alpha;14-3-3 protein beta/alpha, N-terminally processed	Ywhab	Q9CQV8
104	14-3-3 protein epsilon	Ywhae	P62259

Supplementary Table S5. Interaction Domains on Phb2 and their functional relevance.

Phb2 Region (aa)	Interacts with	Functional significance	Reference
120-232	Akt2	Promotes MyoD transactivation function	[42]
120-232	MyoD	Inhibits MyoD transactivation function	[42]
175-198	Estrogen receptor α (ERα)	Represses ERα – mediated transcription	[38]
180-232	mDia1	Regulates MyoG expression	This study

Supplementary Table S6. Antibodies used in this study.

Antibody	IP (µg)	Dilution (IF)	Dilution (Western blot)	Type	Cat. number	Company
mDia1	3	-	1:1000	Monoclonal	610849	BD transduction laboratories
mDia1	-	1:400	-	Polyclonal	-	Raised in lab against mDia1ΔN3
Phb1	-	-	1:1000	Polyclonal	2426S	Cell signalling technology
Phb2	-	-	1:20,000	Polyclonal	sc-67045	Santa Cruz

Phb2	3	-	-	Polyclonal	LS-C287526	LSBio
Phb2	3	1:100	-	Monoclonal	H00011331-M02	Novus Biologicals
pPhb2 Tyr128	-	-	1:500	Polyclonal	AP7270d	Abgent
pPhb2 Tyr248	-	-	1:500	Polyclonal	AP7270e	Abgent
Akt1	-	-	1:1000	Polyclonal	2967L	Cell signalling technology
Akt2	-	-	1:10,000	Polyclonal	3063S	Cell signalling technology
pAkt2(ser474)	-	-	1:1000	Polyclonal	8599S	Cell signalling technology
MyoD	-	1:100	1:1000	Monoclonal	M3512	Dako
β-actin	-	-	1:500	Polyclonal	ab8227	Abcam
GAPDH	-	-	1:2000	Monoclonal	ab9484	Abcam
MyoG	-	1:200	1:1000	Monoclonal	sc-12732	Santa Cruz
Flag	3	-	1:4000	Monoclonal	F3165	Sigma
Flag	-	1:500	-	Polyclonal	F7425	Sigma
GFP	-	-	1:4000	Polyclonal	ab6556	Abcam
GFP	-	1:200	-	Polyclonal	A10262	Thermofisher Scientific
Active β-Catenin	-	-	1:1000	Monoclonal	05-665	Millipore
LaminA/C	-	-	1:5000	Polyclonal	ab58529	Abcam
Lamin B1	-	-	1:5000	Polyclonal	ab16048	Abcam
Peroxidase anti-mouse IgG	-	-	1:5000	Polyclonal	115-035-166	Jackson ImmunoResearch
Peroxidase anti-rabbit IgG	-	-	1:5000	Polyclonal	711-035-152	Jackson ImmunoResearch
Rabbit IgG	3	-	-	Polyclonal	12-370	Millipore
Mouse IgG	3	-	-	Polyclonal	12-371	Millipore

Alexa fluor anti-rabbit 568	-	1:500	-	Polyclonal	A10042	Thermofisher Scientific)
Alexa fluor mouse 647	-	1:500	-	Polyclonal	A-31571	Thermofisher Scientific
Alexa fluor chicken 488	-	1:1000	-	Polyclonal	A11039	Thermofisher Scientific

Supplementary Figure S1.

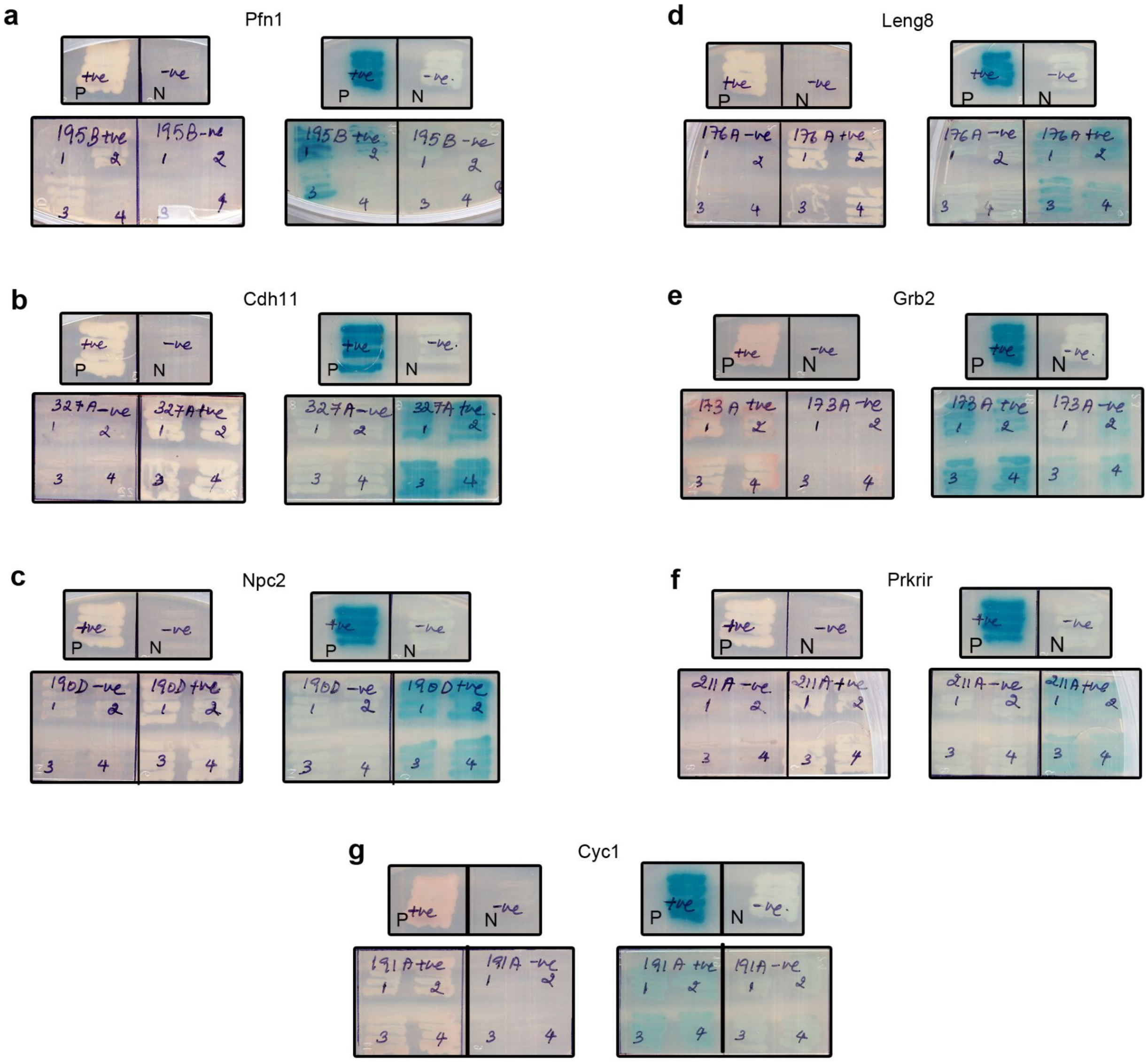

Supplementary Figure S2.

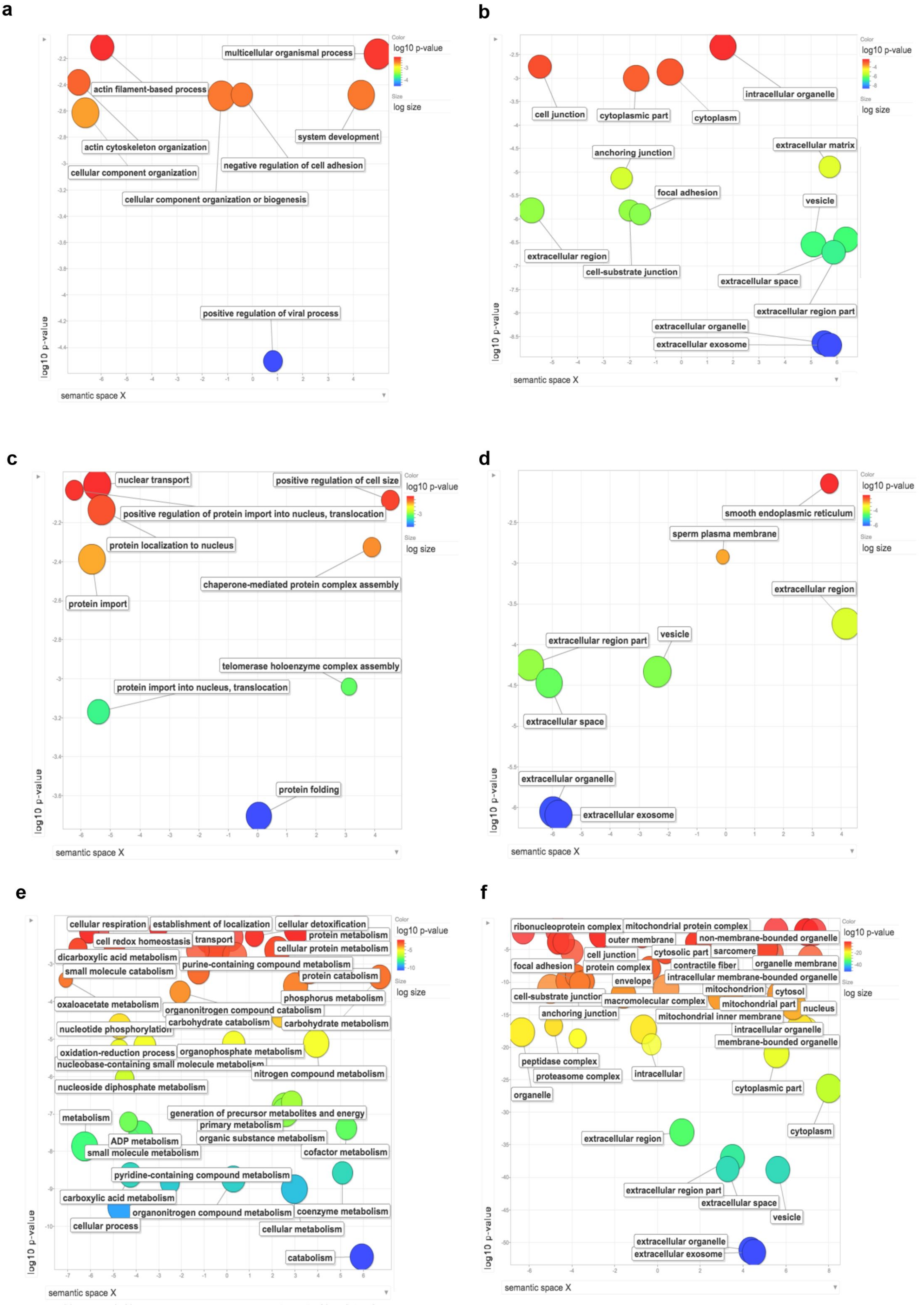

Supplementary Figure S3.

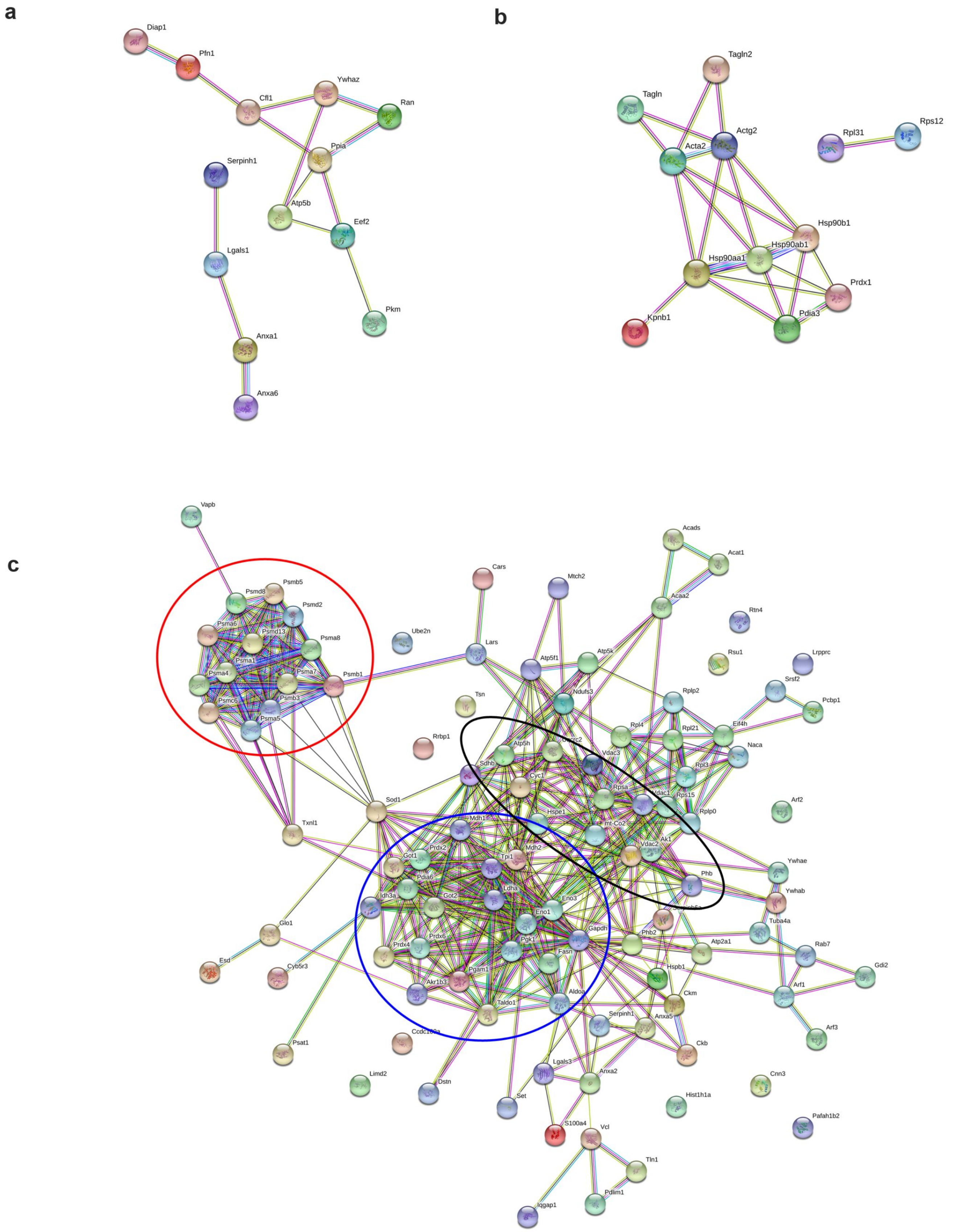

Supplementary Figure S4.

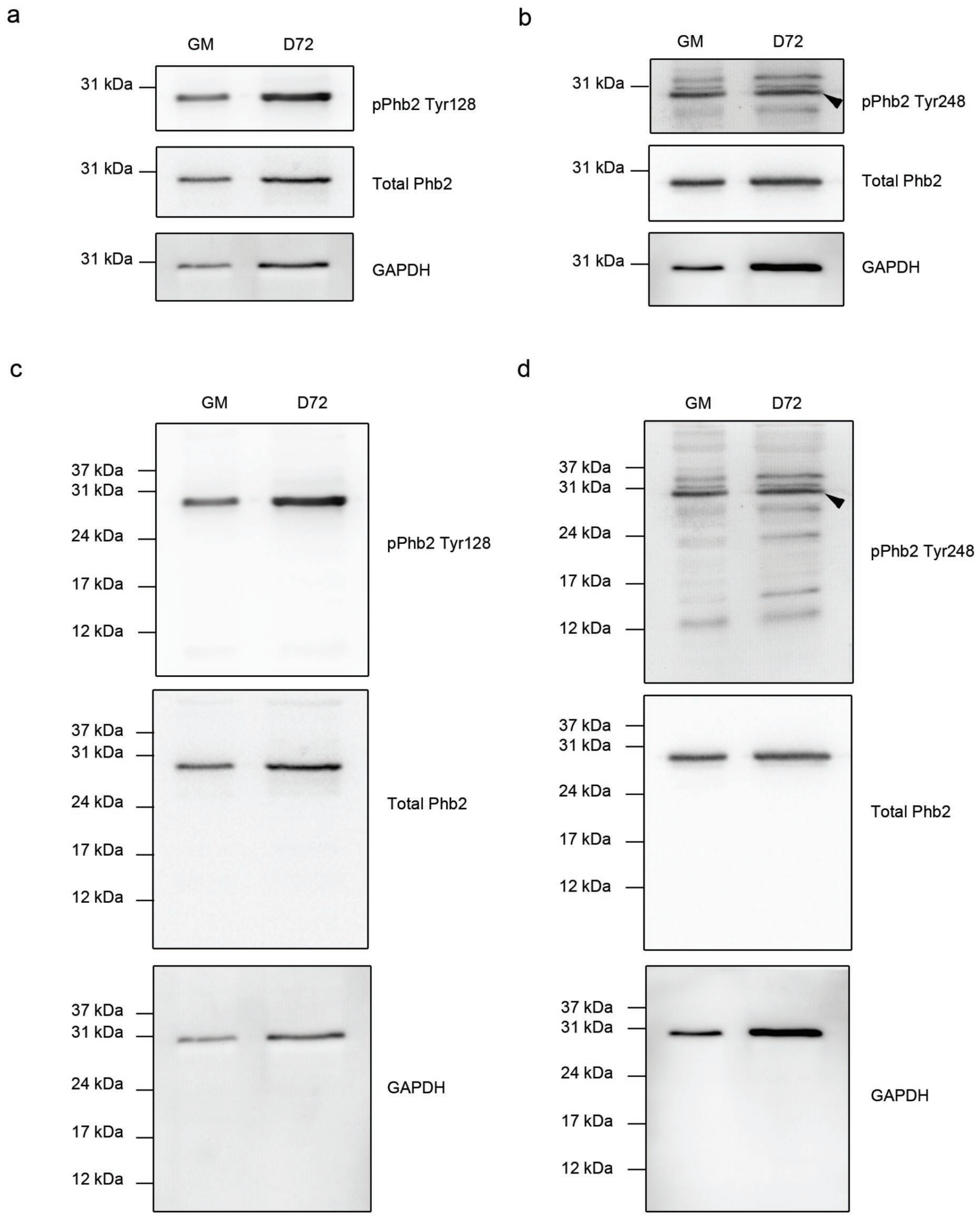

Supplementary Figure S5.

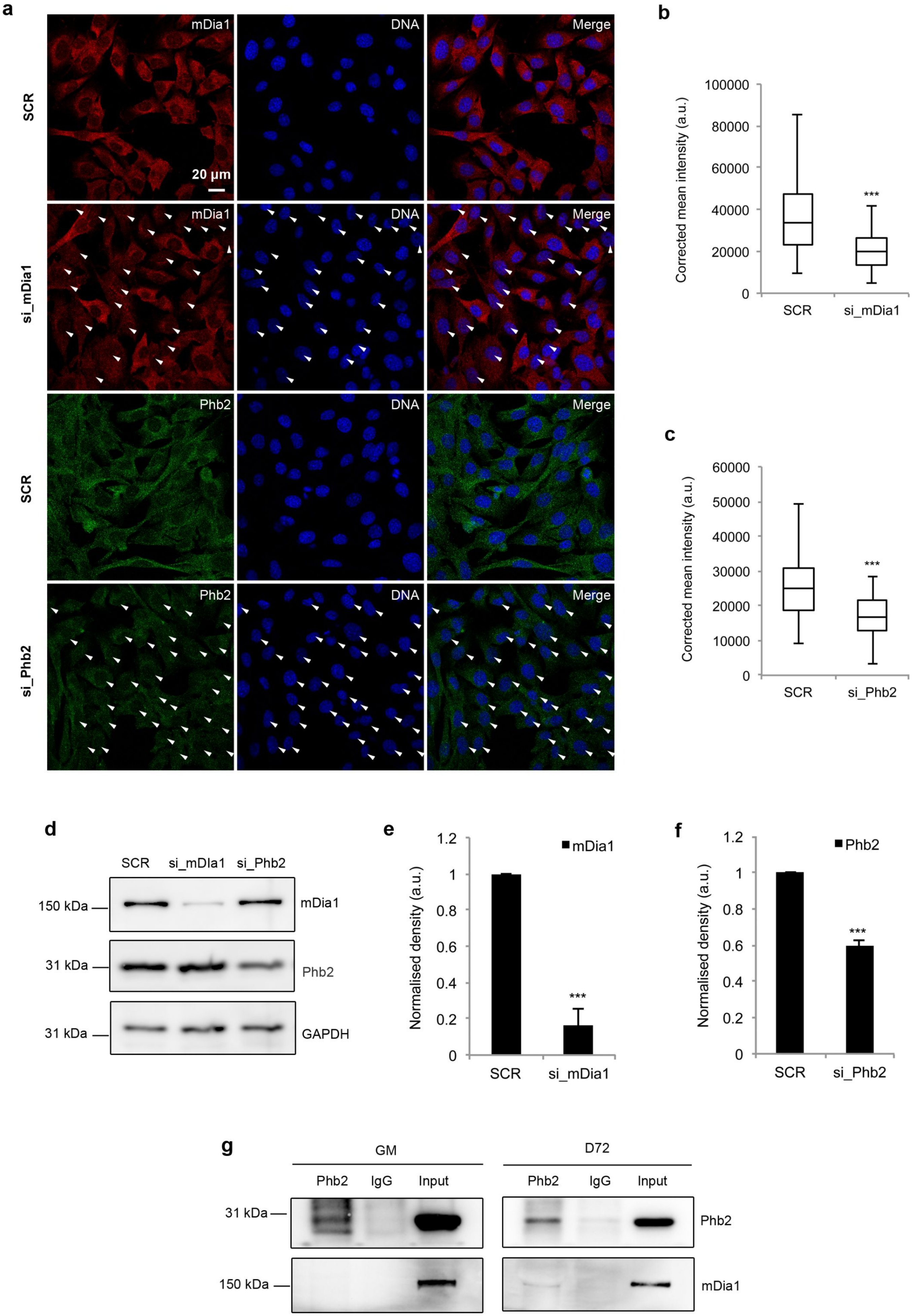

Supplementary Figure S6.

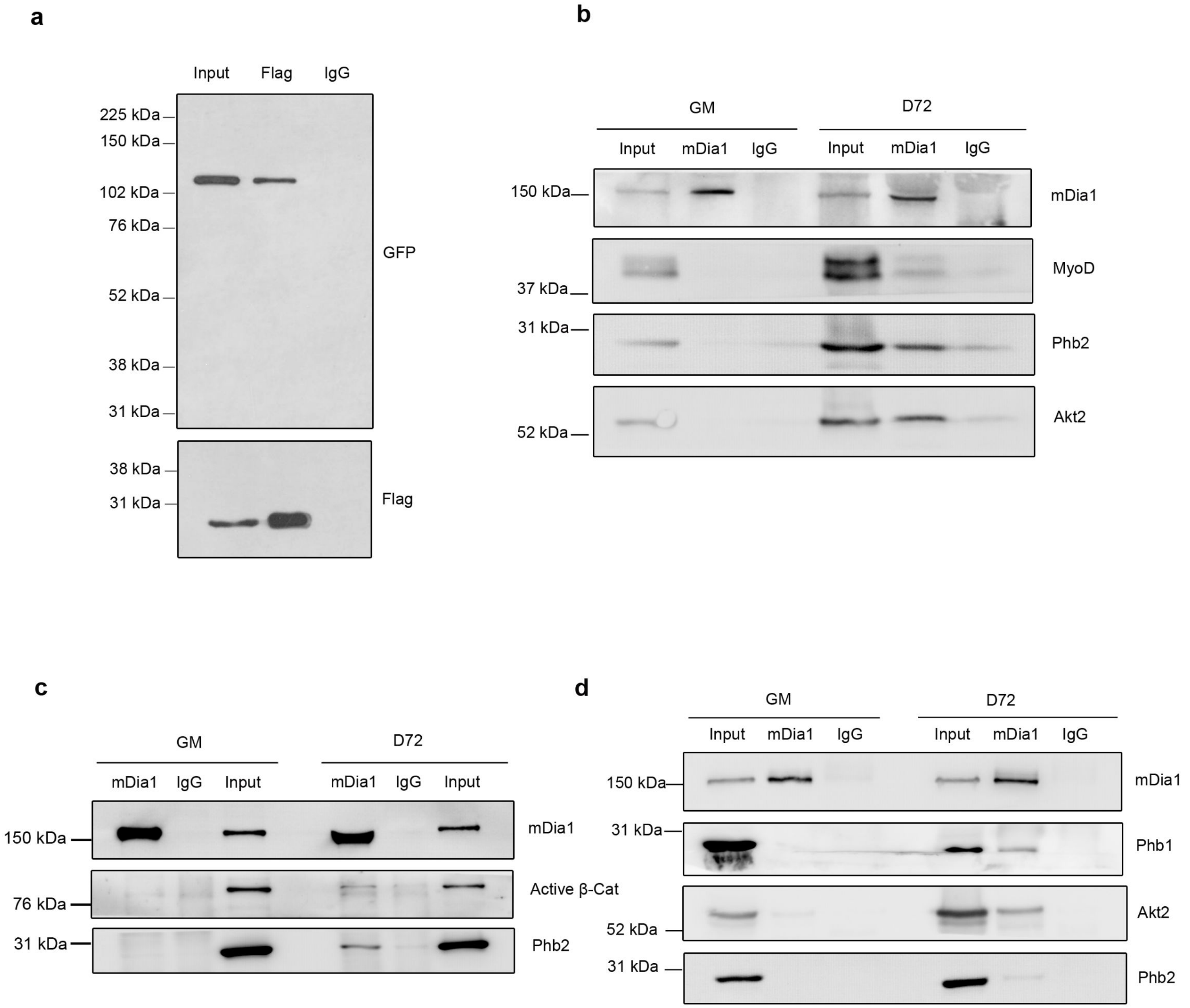

Supplementary Figure S7.

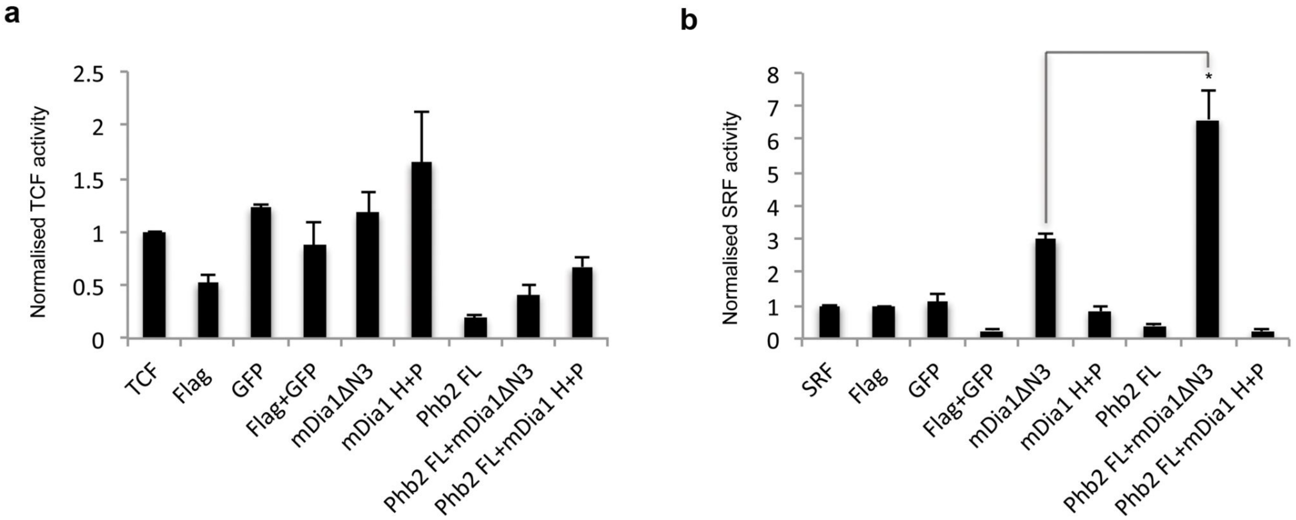

Supplement: Supplementary file 1 — Supplementary Information [file 41598_2019_44749_MOESM1_ESM.pdf]
